# Magnetic hyperthermia as an intracellular immunomodulatory technology: From heat transduction to tumor immune reprogramming

**DOI:** 10.7150/thno.130650

**Published:** 2026-06-25

**Authors:** Bin Yan, Lijun Dai, Hugang Li, Kexin Yuan, Huimin Yao, Lidan Chang, Haibin Peng, Xing-Jie Liang, Xiaoli Liu

**Affiliations:** 1 Department of Hepatobiliary Surgery, The First Affiliated Hospital of Xi'an Jiaotong University, Xi'an, Shaanxi, 710061, China.; 2 National Local Joint Engineering Research Center for Precision Surgery & Regenerative Medicine; Shaanxi Province Center for Regenerative Medicine and Surgery Engineering Research; Shaanxi Provincial Key Laboratory of Magnetic Medicine; First Affiliated Hospital of Xi’an Jiaotong University, Xi’an, Shaanxi, 710061, China.; 3 Institute of Regenerative and Reconstructive Medicine, Med-X Institute, First Affiliated Hospital of Xi’an Jiaotong University, Xi’an, Shaanxi, 710049, China.; 4 Laboratory of Resource Biology and Biotechnology in Western China, Ministry of Education; Provincial Key Laboratory of Biotechnology of Shaanxi Province, Northwest University, Xi’an, Shaanxi, 710069, China.; 5 School of Future Technology, Xi’an Jiaotong University, Xi’an, Shaanxi 710049, China.; 6 CAS Key Laboratory for Biomedical Effects of Nanomaterials and Nanosafety, Chinese Academy of Sciences and National Center for Nanoscience and Technology of China, Beijing 100190, China.

**Keywords:** magnetic hyperthermia, magnetic nanoparticles, drug delivery, immunotherapy, anticancer therapy

## Abstract

Magnetic hyperthermia (MH) has evolved from a localized thermal modality into an emerging intracellular immunomodulatory technology for cancer treatment. By employing magnetic nanoparticles (MNPs) to convert alternating magnetic fields into confined intracellular heat, MH enables precise spatial and temporal regulation of cellular stress responses while maintaining favorable biocompatibility and tumor selectivity. This review highlights recent advances that reposition MH as a cell-level immune regulation strategy, focusing on three interconnected aspects: (i) rational design of MNPs to optimize magnetic-to-thermal conversion efficiency and achieve precise intracellular heat transduction; (ii) integration of magnetic field–controlled MH with drug delivery and nano-heating modalities to enable synergistic intracellular regulation; and (iii) mechanistic insights into MH-induced immune modulation, including immunogenic cell death, immune cell activation, and its integration with tumor vaccines and cancer immunotherapy. Collectively, these advances establish MH as a versatile intracellular immunoregulatory platform and underscore its potential for precision cancer immunotherapy.

## 1. Introduction

Magnetic hyperthermia (MH) was first proposed by Gilchrist *et al.* in 1957 as a therapeutic modality for cancer treatment 1. This approach employs magnetic iron oxide nanomaterials (MIONs) that, when internalized by cells such as tumor cells and subsequent exposure to an alternating magnetic field (AMF), generate localized nanoscale heating (41-44 ºC) and reactive oxygen species (ROS) 2. MH can directly and selectively induce physical thermal killing to tumor cells, while simultaneously eliciting a range of biological effect that further regulate tumor cells fate. We specifically refer to this uniquely advantageous form of thermal therapy occurring within cells as intracellular MH. Compared with conventional thermal modalities such as microwave and radiofrequency ablation, intracellular MH exhibits the distinct advantages of the superior tissue penetration and subcellular level controllability 3. Moreover, the ability of AMF to penetrate tissue without depth limitation, combined with advances in nanotechnology, has significantly propelled the clinical advancement of MH, particularly in the treatment of gliomas and prostate cancer 1, 4-6. Despite these advancements, several challenges remain in the clinical implementation of MH: (1) low efficiency of magnetic nanoparticles (MNPs) in converting magnetic energy to heat, necessitating higher doses; (2) poor delivery and uneven distribution of thermotherapy nano-agents and anti-tumor nano-drugs within tumors, which compromise therapeutic efficacy; (3) the immune-modulating effects of MH that require further investigation.

To address these challenges, several strategies have been developed. In consideration of the electromagnetic safety of AMF, the magnetic field intensity (*H*) and frequency (*f*) to which patients may be exposed should be controlled within *H* × *f* ≤ 5 × 10^9^ A m^-1^ s^-1^ 2. As such, the therapeutic efficacy of MH mainly depends on the magnetothermal conversion efficiency of the MNPs themselves 7, 8. Superparamagnetic iron oxide nanoparticles (SPIOs), favored for their high biocompatibility 9, 10, are commonly used in clinical settings. However, their limited magnetothermal conversion due to small size and low magnetic properties makes them less effective in clinical applications 11. Research efforts are thus dedicated to improving the MH performance of SPIOs through modifications in particle size 12, 13, composition 14, 15, morphology 16-19, and surface characteristics 20-24. In addition to the intrinsic magnetothermal conversion efficiency of MNPs, their intra-tumoral distribution within the tumor microenvironment (TME) critically influences the overall MH efficacy. Under an applied AMF, MNPs not only generate localized hyperthermia but also undergo spatial displacement driven by magnetic field. Consequently, MNPs-based drug delivery systems can facilitate the transport of therapeutics across multiple physiological barriers—including vascular walls, dense tumor stroma, and cellular membranes. Even in regions characterized by elevated interstitial fluid pressure, such systems markedly enhance drug penetration depth and intra-tumoral distribution uniformity 25. Moreover, integrating active drug penetration *via* magnetic field with precise intracellular release triggered by magnetothermal stimulation represents a promising strategy to further improve MH efficacy 26. At the level of immune regulation, accumulating evidence indicates that MH can regulate the physiological and biochemical properties of both tumor cells and immune cells, altering their biological functions 27. These alterations contribute to amplifying anti-tumor immune responses, for example, activation of the immune system through ROS-dependent immunogenic cell death (ICD) 28, promotion of macrophages polarization toward the pro-inflammatory M1 phenotype 29, dendritic cells (DCs) maturation 30, and accelerated T lymphocyte infiltration into tumor sites 31. Further exploration of these biological effects and regulatory mechanisms at the cellular and subcellular levels is necessary to fully leverage MH in enhancing tumor immunotherapy responsiveness.

**Scheme 1:**
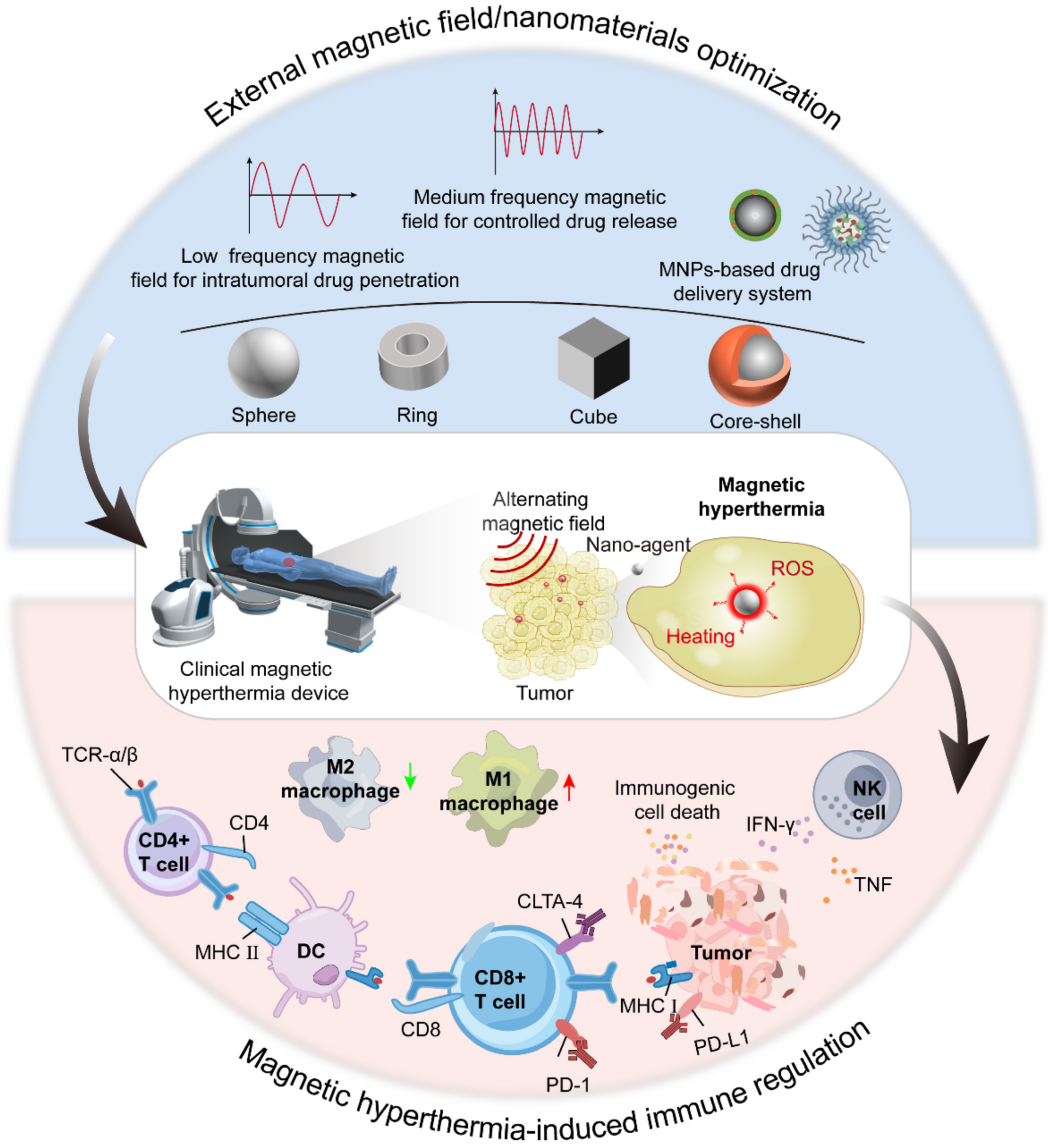
Schematic illustration of strategies to enhance MNPs-mediated MH therapy. This includes (1) Optimization of MNPs performance and AMF parameters to maximize magnetothermal therapeutic efficiency; (2) MH-enabled, MNPs-mediated intra-tumoral drug delivery and controlled release to improve treatment outcomes; and (3) an in-depth understanding of MH-induced immune regulation, thereby enhancing the efficacy of antitumor immunotherapy.

In this Review, we highlight recent advances that reposition MH from a conventional thermal ablation modality to an intracellular immune regulatory technology (Scheme 1). We first summarize how the rational design of MNPs governs intracellular heat transduction efficiency. We then discuss magnetic field–assisted delivery and magnetothermal-controlled intracellular regulation strategies that enable precise modulation of cellular responses. Finally, we focus on the immunological consequences of MH, emphasizing its roles in immune activation, tumor vaccination, and synergistic integration with cancer immunotherapy. Collectively, these advances establish MH as a versatile platform for cell-level immune modulation and precision cancer theranostics.

## 2. The basic principles of MH

For the successful clinical translation of MH, MNPs must combine high heating efficiency with favorable biosafety profiles. In MH, MNPs act as nanoscale magnetothermal transducers that convert the energy of an externally applied AMF into localized heat 2. Upon exposure to an AMF, the magnetic moments (*m*) of MNPs tend to align with the oscillating field. The kinetics of this magnetization process and the resulting heat generation are jointly determined by the intrinsic properties of the nanoparticles—including particle size, shape, composition, crystallinity, and surface chemistry—as well as by the AMF parameters, namely the *f* and *H*. These magnetothermal behaviors are classically described by the magnetic hysteresis loop, which reflects the relationship between magnetization (*M*) and the applied magnetic field. Key hysteretic parameters include saturation magnetization (*M*_s_), remanent magnetization (*M*_r_), and coercivity (*H*_c_) 32. Importantly, the area enclosed by the hysteresis loop corresponds to the energy dissipated as heat per magnetization cycle and represents the fundamental physical origin of heat generation in MH (Table 1).

The specific heat absorption rate (SAR), also known as the specific loss power (SLP) 2, is a critical metric for assessing the magnetic heating efficiency of MNPs under an applied AMF. To lower the injected MNPs dose and the treatment duration, the SAR values should be as high as possible and some of them reported in the literature even beyond 10,000 W g^-1^ 44. The SAR is calculated using the formula:



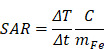



Where *C* represents the specific heat capacity of the solution, *m*_Fe_ represents the mass of iron concentration in the solution, and *ΔT/Δt* represents the initial slope of the temperature-time curve.

The SAR is influenced by the AMF’s *f* and *H*, it is not suitable for comparing results across different AMF settings. In contrast, intrinsic loss power (ILP), which is not affected by magnetic field parameters, providing a consistent measure of a material’s magnetic-thermal conversion efficiency under varying external field conditions. The SAR can be normalized to the ILP using the formula:



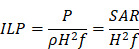



Where *P* denotes the volume power and *ρ* represents the density of the nanomaterials.

Based on magnetic relaxation and hysteresis mechanisms, the heating efficiency of MNPs generally increases with increasing AMF *f* and *H* 45. However, to ensure clinical safety, the product of field amplitude and frequency must remain below a biological tolerance threshold in order to minimize excessive tissue heating arising from induced eddy currents. Such nonspecific Joule heating is independent of nanoparticle-mediated losses and can be further exacerbated by coil-related heating and the presence of conductive implants. Although a universally accepted safety limit has not yet been formally established, early guidelines proposed a conservative criterion of *H* × *f* ≤ 4.85 × 10^8^ A m^-1^ s^-1^ 46, 47. In clinical practice, however, magnetic induction systems such as the M5 device can operate within substantially higher yet still tolerable ranges (*H* × *f* < 5 × 10^9^ A m^-1^ s^-1^). Notably, clinical trials reported by Jordan et al. in patients with glioblastoma demonstrated therapeutic efficacy at *H* × *f* values of approximately 1.6-4.2 × 10^9^ A m^-1^ s^-1^ 48.

These discrepancies highlight that clinically acceptable operating windows depend not only on electromagnetic parameters but also on treatment configuration and control strategies. To mitigate nonspecific heating while preserving therapeutic efficacy, several practical approaches have been adopted, including: (i) selection of moderate frequencies to limit eddy-current losses in tissues; (ii) intermittent AMF operation to reduce cumulative thermal burden; (iii) tumor-targeted nanoparticle delivery and dose minimization enabled by high SAR agents to spatially confine heating; and (iv) integration of real-time temperature monitoring and feedback-controlled field modulation. Collectively, these observations suggest that, under carefully controlled conditions, the clinically acceptable operating window of *H* × *f* may be broader than initially anticipated from theoretical constraints.

## 3. Optimizing MNPs to improve magnetic thermal efficiency

The magnetothermal efficiency of MNPs is commonly evaluated using the SAR or ILP, which quantify the ability of MNPs to convert electromagnetic energy into heat under an AMF. To date, NanoTherm^®^ remains the only metal-based nanomaterial approved by the U.S. Food and Drug Administration (FDA) for MH-based cancer therapy. NanoTherm^®^ is formulated as a colloidal suspension of iron oxide nanoparticles with an average diameter of approximately 15 nm, stabilized by an aminosilane coating 11. In the first clinical application of NanoTherm^®^-mediated MH in patients with prostate carcinoma, a SAR of approximately 288 W kg^-1^ was reported under an AMF (*f* = 100 kHz, H = 0-15 kA m^-1^) 49. However, this relatively limited heating efficiency, together with the heterogeneous intra-tumoral distribution of nanoparticles, has constrained therapeutic outcomes, underscoring the need for further optimization of MNPs design to facilitate clinical translation 50.

In addition to the *f* and *H* of the applied AMF, the SAR and ILP of MNPs are strongly influenced by particle size, shape, composition, crystal structure and magnetic anisotropy and surface functionalization 51. These attributes modulate *M*_s_, the magnetic crystalline anisotropy (*K*), and the Néel/Brown relaxation times, therapy altering the prevailing energy dissipation mechanisms. In general, SAR increases with *M*_s_ and high mono-dispersity (narrow size distribution) favors enhanced SAR value. However, the dependence on absolute particles size and *K* is typically non-monotonic for a given *K* and environmental condition there is often an optimal particles size that maximizes SAR. Moreover, magnetic interactions, aggregation state, medium viscosity and measurement conditions substantially affect the measured SAR/ILP values (Table 2). Consequently, MNPs that exhibit high *M*_s_ and high mono-dispersity are preferred for maximizing magnetic thermal efficiency 52-54.

Meanwhile, conductive nanostructures and magnetoconductive hybrids represent another important mechanism for heat generation. In conventional bulk materials, eddy currents are a parasitic loss that limits transformer efficiency; at the nanoscale, however, they can be harnessed to produce localized heat. This strategy is especially effective when conductive nanostructures or magnetoconductive hybrids are used, with their electrical conductivity, geometry, and field parameters co-optimized to maximize ohmic heating under AMF (Table 3).

The key distinction from MNP-based heating lies in the mechanism: Néel/Brownian relaxation requires superparamagnetic or ferromagnetic cores with specific anisotropy, while eddy-current heating is primarily governed by electrical conductivity and geometry. This opens the door to non-magnetic materials or magnetoconductive hybrids where the conductive component provides an additional, often dominant, heating pathway that can be tuned independently of magnetic hysteresis (Table 4).

### 3.1 Size modulation of MNPs

The size of MNPs is a critical determinant of their magnetic behavior. For bulk MNPs, their magnetic properties are determined by the magnetic structure formed of magnetic domains separated by domain walls and they usually exist in a multi-domain state 63. The *m* of each domain is randomly orientated inside the MNPs in the absence of an external field. When exposed to an applied AMF, these domains align with the field direction, and the magnetization of MNPs reaches saturation. The energy barrier needed to invert the magnetic spin of domains is size-dependent 64. Usually, increasing the size of MNPs improves their magnetic properties such as magnetic susceptibility and *M*_s_ because of increased *K*, suggesting that larger MNPs exhibit higher SAR value 65. However, poor *in vivo* cycling, cell endocytosis and unsatisfactory tumor permeability make MNPs of large size unsuitable for MH.

**Figure 1:**
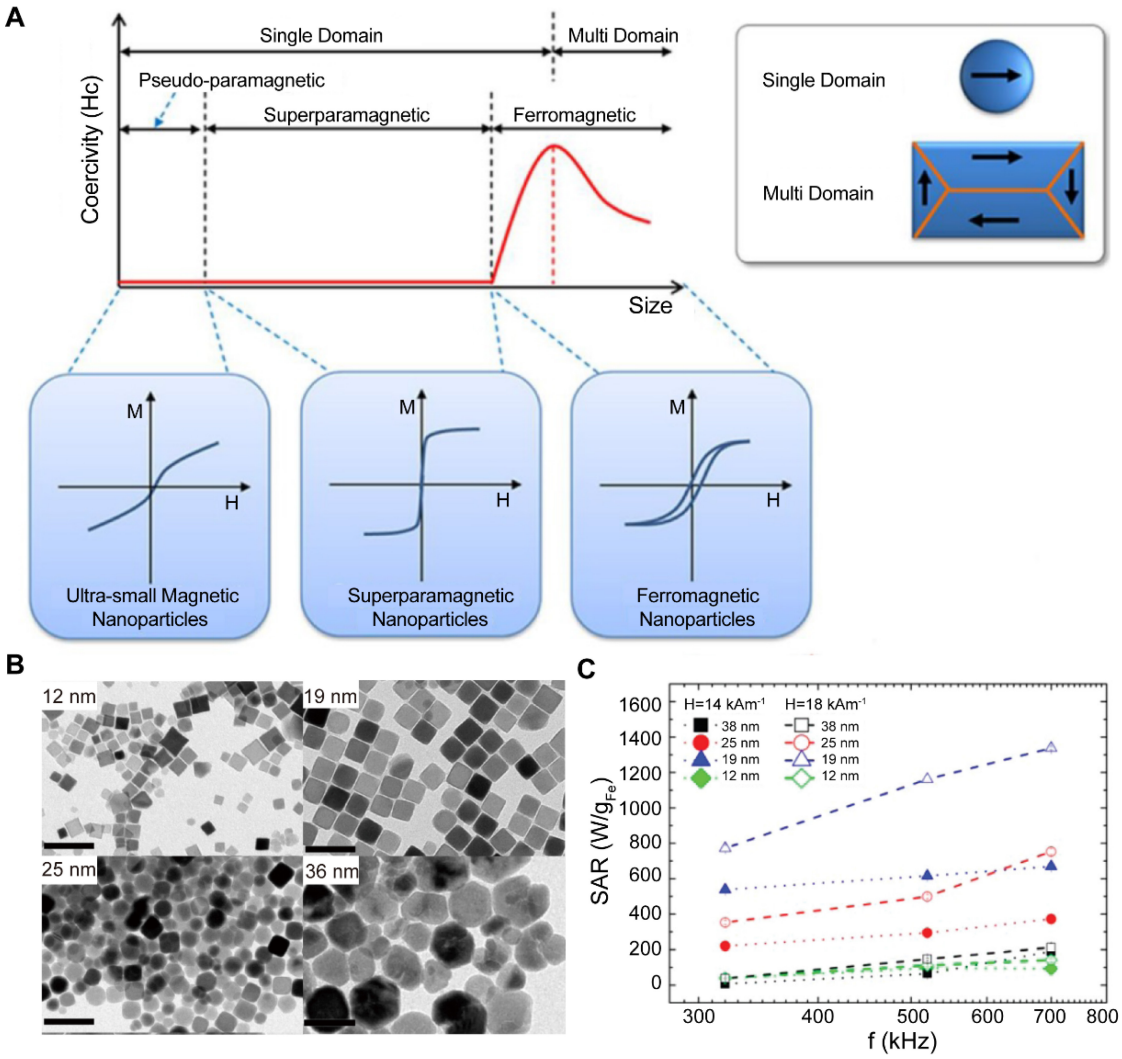
Magnetothermal mechanisms and size-dependent effects of MNPs. (A) Néel and Brownian relaxation mechanisms. Adapted with permission from 2. Copyright 2020, Ivyspring International Publisher. (B) Representative transmission electron microscopy (TEM) images of cubic iron oxide nanocrystals. Scale bar = 100 nm. (C) SAR values as a function of frequency and nanoparticle size. Adapted with permission from 69. Copyright 2012, American Chemical Society.

Below a critical size, the formation of a single-domain state becomes energetically favorable. As particle size decreases further, MNPs change from multi-domain ferromagnetism to single-domain ferromagnetism, and finally to superparamagnetism. Multi-domain MNPs retain remanent *M*_r_ even after removal of the external field and require a further reverse magnetic field *H*_c_ for complete demagnetization. This hysteretic behavior creates the source of heat in MH 66. Single-domain MNPs show the largest *H*_c_ and Hysteresis losses due to a high energy barrier for spin inversion, whereas superparamagnetic particles combine high *M*_s_ with negligible *H*_c_ and *M*_r_, resulting in a hysteresis loop of nearly zero area (Figure 1A). For SPIOs, heat generation in AMF arises predominantly from Néel and Brown relaxation losses 67. Néel relaxation is the rotations needed to overcome the friction between magnetization easy axis and atomic lattices and Brownian relaxation is for MNPs and their surroundings 68. SPIOs show promising clinical availability given that they are inert in the absence of the AMF but they could response to *H* easily once the AMF is applied.

Particle size governs the SAR by modulating saturation magnetization and magnetic anisotropy. For SPIOs, *M*_s_ generally increases with increased size, which explains the low SAR values (~300 W/g) typically observed for conventional polydisperse SPIOs. Guardia *et al.* prepared cube-shaped iron oxide nanocrystals (IONCs) by thermal decomposition method that ranged in size from 13 to 40 nm (Figure 1B, C) 69. The SAR value of the IONCs with an average size of 19 nm was 2,452 W/g (*f* = 520 kHz, *H* = 29 kA m^-1^), which was one of the highest values for IONCs. In contrast, IONCs prepared by sol-gel or co-precipitation methods have shown a considerable polydispersity, which could compromise their SAR values. On the other hand, there is a lack of standard measurement protocol and many of the reported SAR values were measured at *f* from 500 to 700 kHz and *H* from 10 to 20 kA m^-1^, leading to the variability of SAR values. Guardia 69 and Hergt 70 reported that particles near the transition between superparamagnetic and ferromagnetic behavior (~20 nm for iron oxide) exhibit maximal SAR/SLP value. However, the precise boundaries of this transition region remain ambiguous, with reported ranges spanning 2 to 800 nm depending on particle shape, crystallinity, and interfacial effects 71. Collectively, the most promising designs for MH involve highly crystalline, mono-disperse iron oxide nanoparticles positioned within the magnetic transition regime. This size range provides an optimal balance, maximizing magnetic losses through enhanced *M*_s_ and *K*.

### 3.2 Shape anisotropy of MNPs

**Figure 2:**
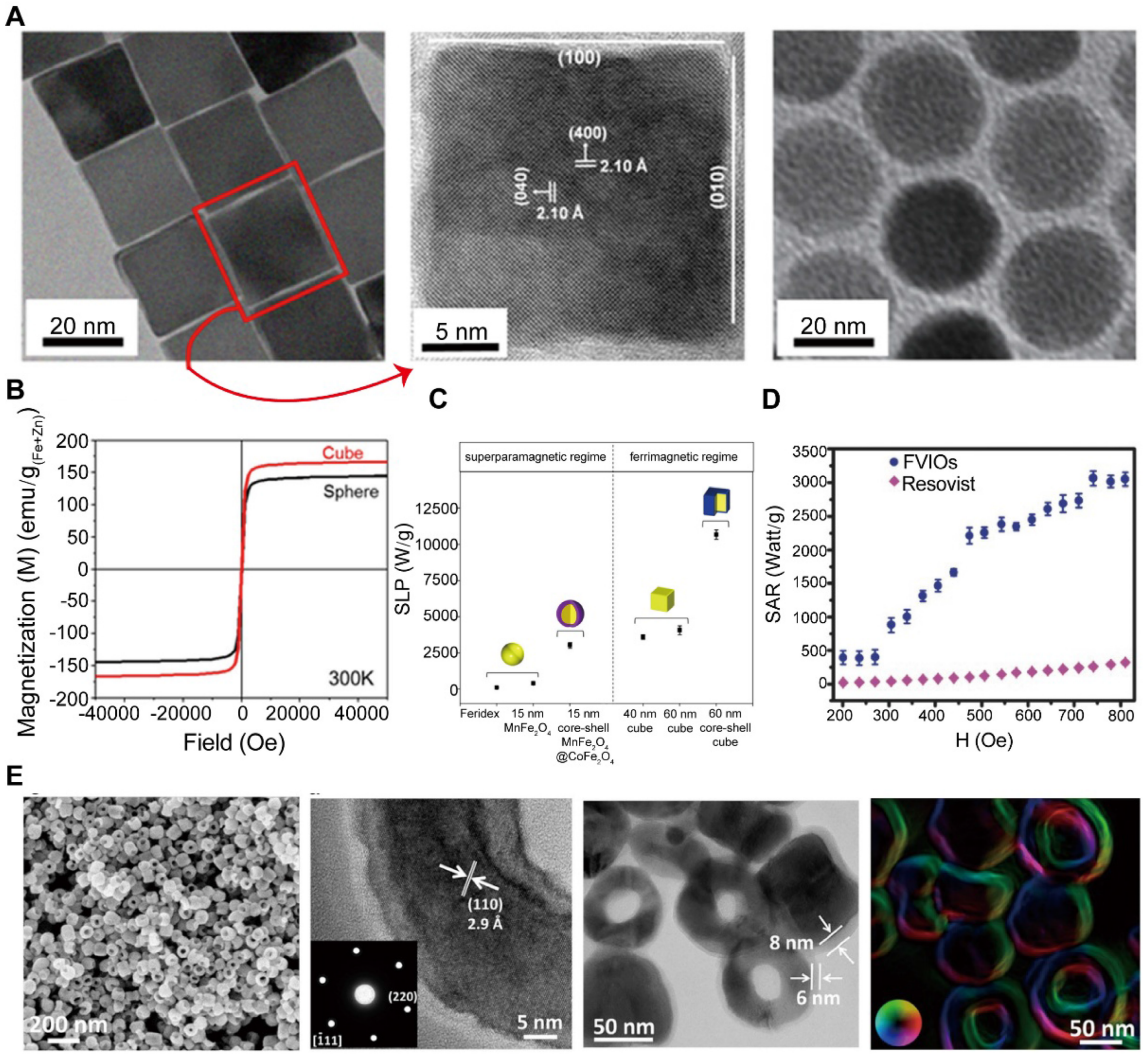
Influence of nanoparticle shape on magnetic properties and heating performance. (A) Representative TEM images and corresponding magnetization curves at 300 K for cubic and spherical nanoparticles with identical chemical composition and magnetic volume. (B) Comparison of the magnetization behavior of cubic and spherical nanoparticles, highlighting the higher saturation magnetization exhibited by cubic particles. (C) Summary of SLP values for various nanoparticle systems, showing that core–shell cubic nanoparticles achieve the highest SLP (10,600 W/g). Adapted with permission from 73. Copyright 2012, American Chemical Society. (D) Comparison of SAR values of FVIOs and Resovist under different magnetic field amplitudes at a fixed frequency (*f* = 400 kHz). (E) Characterization of the morphology, crystal structure, and surface modification of FVIOs. Adapted with permission from 74. Copyright 2015, John Wiley & Sons, Inc.

Shape anisotropy serves as a key determinant in modulating the magnetothermal performance of MNPs. Different shapes, such as nanospheres, nanocubes, nanotubes, nanoflowers and nanorings, exhibit different shape anisotropy, thereby producing substantial variations in magnetic anisotropy, hysteresis losses, and SAR values 72. Moreover, shape anisotropy not only redistributes the relative contributions of Néel and Brown relaxation to energy dissipation but also alters the configuration of surface spins, collectively shaping the overall magnetic heating efficiency. Noh *et al.* found that Zn_0.4_Fe_2.6_O_4_ in the cubic form exhibited a higher *M*_s_ and better heat production performance than in the spherical form, which might be attributed to the 50% reduction in the proportion of spin disordered layers on the cubic surface 73. Further encapsulation of Zn_0.4_Fe_2.6_O_4_ into a CoFe_2_O_4_ shell by exchange anisotropy, resulted in a hybrid nanocube with a 14-fold increase in the *H*_c_ and a SAR value as high as 10,600 W/g (*f* = 500 kHz, *H* = 37.4 kA m^-1^) (Figure 2A-C). The exchange anisotropy at the core-shell interface significantly enhances hysteresis heating by increasing the effective anisotropy and *H*_c_, but this enhancement is often accompanied by a higher residual magnetism and the risk of long-term retention *in vivo*. In contrast, planar dominant sharp such as cubic and octahedral mainly enhance *M*_s_ by reducing surface spin disorder, indicating that regulating the surface spin structure is another independent approach to improving magnetization ability.

Liu *et al.* reported that ferrimagnetic vortex-domain iron oxide nanorings (FVIOs) exhibited negligible remanence and large hysteresis loss during MH 74. The *m* of FVIOs is distributed in a vortex in the direction of clockwise or counterclockwise, forming a unique multi-domain structure with a closed distribution of magnetization. Because magnetization was circumferential without stray fields, FVIOs showed comparable dispersity with SPIOs and external field-induced vortex-onion state magnetization reversal. The SAR of FVIOs could reach 3,050 W/g (*f* = 362 kHz, *H* = 24.5 kA m^-1^) (Figure 2D, E), therefore only a very low dose of 0.3 mg/cm^3^ was needed for MH of MCF-7 breast cancer tumor-bearing nude mice, which was much less than previous reported doses of 5-10 mg/cm^3^ to elicit MH in mouse breast cancer tumor tissue.

In summary, shape engineering represents a powerful approach to tuning magnetothermal performance. Polyhedral morphologies tend to suppress surface spin disorder, thereby increasing the effective saturation magnetization and improving per-particle heating efficiency. Exchange-coupled core-shell architectures can markedly enhance coercivity and hysteresis losses; however, such gains are often accompanied by increased remanence and a heightened risk of long-term *in vivo* retention. In contrast, vortex- or ring-like topologies decouple low remanence from large field-induced switching losses, enabling high SAR at ultralow particle doses—an attribute that is particularly desirable for clinical translation.

### 3.3 Advanced MNPs components

Beyond size and shape modulation, compositional tuning through divalent transition-metal cation doping constitutes a fundamental strategy for enhancing magnetothermal performance. Mixed-metal ferrites with an M_x_Fe_3₋x_O_4_ structure typically exhibit increased saturation magnetization, magnetic anisotropy, and magnetic susceptibility, leading to superior heating performance 75, 76. This enhancement originates from lattice-level modulation of electronic configurations and superexchange interactions induced by the dopant cations, which collectively strengthen magnetic ordering and promote energy dissipation.

Jang *et al*. reported a biocompatible γ-Fe_2_O_3_ system lightly doped with magnesium 77. The controlled distribution of Mg^2+^ cations enhance the out-of-phase component of magnetic susceptibility and magnetic softness, resulting in an ILP of up to 14 nH m^2^ kg^-1^—approximately two orders of magnitude higher than that of the commercial MRI contrast agent Feridex. The magnetothermal potential of this material was further corroborated in both *in vitro* and *in vivo* models. Nevertheless, compositional tuning is rarely a linear optimization strategy and generally necessitates simultaneous control over surface and interfacial properties to balance enhanced lattice magnetic ordering against suppressed surface spin disorder. To overcome the inherent uncertainty of random doping, Lee *et al*. developed exchange-coupled core-shell nanoparticles composed of magnetically hard and soft phases, enabling fine regulation of magnetic anisotropy 78. These nanoparticles exhibited SLP values in the range of 1,000-4,000 W/g, substantially outperforming single-component cobalt ferrites or soft magnetic materials and exceeding Feridex by more than 30-fold. The superiority of such exchange-coupled architectures arises from their engineered interfaces, which synergistically integrate the high saturation magnetization of the soft phase with the large anisotropy of the hard phase, providing a more controllable and reproducible pathway to high-performance MH than compositional doping alone.

Beyond metal doping and exchange-coupled core–shell architectures, other heterogeneous nanostructures have shown substantial potential. For example, Ag/Fe_3_O_4_ nanoflowers can mediate synergistic therapies combining MH and photothermal therapy 79, while IONF@CuS nanohybrids facilitate enhanced magnetothermal-photothermal effects over a broader spectral range 80. Furthermore, Au-Fe_x_O_y_ Janus dimers can be engineered as smart, multifunctional theranostic agents 81. Overall, mixed-metal ferrites improve magnetic ordering *via* lattice-level compositional tuning, and lightly doped systems have already demonstrated markedly enhanced ILP and *in vivo* efficacy. Concurrently, exchange-coupled core-shell architectures offer precisely tunable and reproducible magnetic anisotropy, achieving SLP that far surpass those of conventional ferrites. Collectively, these advances indicate that next-generation MH agents will increasingly rely on rationally designed, multicomponent architectures that balance high SAR, controllable anisotropy, and multifunctionality, thereby enabling clinically meaningful heating at ultralow particle doses.

### 3.4 Surface modification of MNPs

Surface modification is another pivotal factor affecting the performance of MNPs, as it influences not only colloidal stability and biocompatibility but also directly determines magnetothermal efficiency. Liu *et al*. examined the effects of polyethylene glycol (PEG) coatings of varying molecular weights on the heating efficiency of Fe_3_O_4_ nanoparticles, revealing an inverse relationship between coating thickness and SAR 57. Thicker coatings increase the contribution of Brownian relaxation while potentially hindering thermal conduction, ultimately diminishing magnetothermal conversion efficiency. Notably, 19-nm Fe_3_O_4_@mPEG2,000 nanoparticles exhibited the highest SAR, underscoring the importance of precise optimization of coating thickness to achieve a balance between colloidal stability and heating performance. Another study 82 developed a hybrid nanoplatform comprising FVIOs integrated with graphene oxide (GO). Under a specific AMF, this composite achieved an exceptionally high SAR of 5,054 W/g. Compared with single-component nanoparticles, the hybrid system not only enhanced heating efficiency but also markedly amplified ROS generation. *In vivo* experiments demonstrated effective tumor hyperthermia with only two AMF treatments at an ultralow dose of 3 mg Fe/kg, whereas conventional SPIOs therapies typically require higher doses and multiple treatments.

In summary, surface modification plays a pivotal role in determining the physicochemical stability, magnetothermal efficiency, and functional versatility of nanoparticles. Through rational design of surface coatings or composite architectures, it is possible to concurrently optimize biocompatibility, SAR, and ROS generation, thereby offering an effective strategy for achieving low-dose, high-efficiency MH.

### 3.5 Nanoscale thermometry methods

Existing research evidence suggests that under the influence of an alternating magnetic field, nanoparticles can generate significant localized temperature increases on their surface, while the overall temperature change of the cell remains minimal. However, this conclusion primarily relies on nanoscale temperature measurement techniques, which have notable limitations and controversies. Angel Millán's team reported the real-time variation of the local temperature on γ-Fe_2_O_3_ magnetic nanoheaters using a Sm^3+^/Eu^3+^ ratiometric luminescent thermometer placed on their surface during exposure to an external AMF 83. The results indicated that the surface temperature of the nanoparticles could increase by up to 8 °C under the alternating magnetic field. In contrast, synchronized measurements taken on the cell membrane revealed no significant temperature change. Based on thermodynamic calculations, the total heat generated by the nanoparticles within 5 minutes would only be sufficient to raise the overall temperature of the entire cell by approximately 0.2 °C. Given that these cells are immersed in the cell culture medium, which acts as a large heat sink, the overall temperature increase is physically nearly impossible.

However, this conclusion primarily relies on nanoscale temperature measurement techniques, which have notable limitations and controversies. Current nanoscale thermometry mainly relies on methods such as ratiometric luminescent nanothermometry, X-ray absorption nanothermometry, and phase-transition-based nanothermometry, each of which exhibits typical artifacts and limitations. Although the ratiometric luminescence method enables *in situ* measurements on nanoparticle surfaces, factors such as selective absorption and scattering of excitation/emission light by biological tissues, as well as variations in probe concentration, can distort the spectra and introduce errors as large as several tens of degrees celsius. The X-ray absorption method requires synchrotron radiation sources, involves stringent equipment requirements, and has limited temporal resolution, making it difficult to capture transient thermal processes. Phase-transition-based nanothermometry is constrained by material specificity, facing challenges in complex biological liquid environments. Therefore, conclusions regarding “local hot spots” derived from a single thermometry technique must be interpreted with caution; cross-validation with multiple techniques and integration with heat transfer models are often necessary to reliably distinguish between local heat generation and overall temperature rise.

## 4. MNPs-mediated drug delivery system

MNPs, owing to their combined capabilities for magnetic field–guided navigation and magnetothermal triggered release, have emerged as highly promising platforms for drug delivery. MNPs-based delivery systems can minimize nonspecific drug distribution during systemic circulation and enable preferential accumulation at target sites under external magnetic guidance. Importantly, precise spatial localization and on-demand drug release can be achieved by tuning the parameters of the applied AMF 84-87. By integrating passive targeting mechanisms, such as the enhanced permeability and retention (EPR) effect, with remotely triggered active release, this strategy affords markedly improved spatiotemporal control over drug delivery compared with conventional approaches.

Soleimang *et al*. developed core–shell Cu@Mn_3_O_4_-TMC/5-Fu nanoparticles for magnetically triggered delivery of 5-fluorouracil 88. This nanoplatform exhibited optimal drug release under acidic conditions (pH 5.2) and in the presence of an AMF, with the release efficiency increasing monotonically with magnetic field frequency and reaching approximately 92% at 430 Hz. Notably, even at relatively high concentrations and under magnetic field exposure, the carrier showed minimal cytotoxicity toward normal human mammary epithelial MCF-10A cells, while significantly suppressing the viability of breast cancer MCF-7 cells, indicating favorable biocompatibility and pronounced tumor selectivity. These results demonstrate that pH-responsive behavior and magnetic field–driven mechanisms can synergistically enable TME–adaptive and remotely controllable drug release. Furthermore, the strong frequency dependence of drug release suggests that magnetothermal and/or magnetic field–mediated effects play a dominant role in regulating release kinetics in this system.

Noninvasive, stimulus-responsive delivery systems that integrate magnetic fields with SPIOs enable precise spatiotemporal regulation of drug release. Zink *et al*. engineered core–shell MNPs consisting of a soft-magnetic MnFe_2_O_4_ core and a hard-magnetic CoFe_2_O_4_ shell, whose surfaces were functionalized with a thermosensitive “gatekeeper” molecule (ACVA) to construct a Mag@MSNs-ACVA drug delivery platform 89. At physiological temperature, ACVA effectively sealed the nanopores of the carrier, preventing premature doxorubicin (DOX) leakage and thereby substantially reducing off-target toxicity. In contrast, exposure to an AMF triggered remote and controllable release of DOX, with cytotoxic efficacy being precisely modulated by adjusting the duration of magnetic field application. Collectively, this thermoresponsive gatekeeping strategy underscores the potential of magnetically actuated nanoplatforms for achieving highly controllable and targeted drug delivery in cancer therapy.

**Figure 3:**
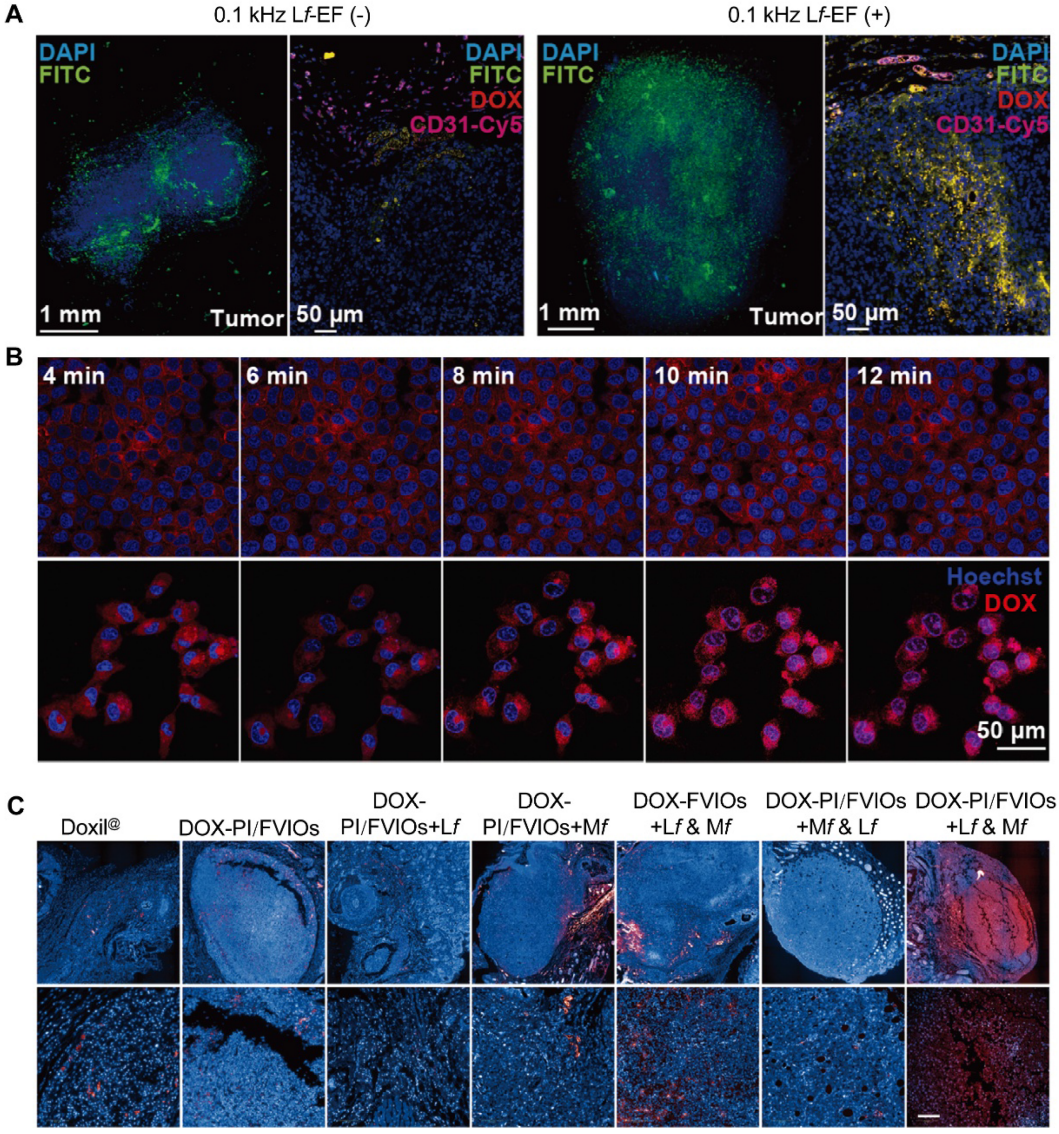
Cascaded drug delivery and release enabled by frequency-switchable electromagnetic fields. (A) Spatial distribution of nanocarriers within three-dimensionally reconstructed tumor tissue and fluorescence analysis of their colocalization with tumor vasculature. (B) Real-time imaging and quantitative analysis of doxorubicin release from the nanocarrier system and its subsequent accumulation in cell nuclei under medium-frequency electromagnetic field stimulation. (C) Representative fluorescence images of tumor sections following different treatment regimens. Adapted with permission from 90. Copyright 2021, John Wiley & Sons, Inc.

Exploiting the frequency-dependent responses of MNPs to alternating electromagnetic fields, an intelligent drug delivery system can be engineered to achieve both efficient tissue penetration and controllable release. Under a low-frequency electromagnetic field (L*f*-EF), MNPs primarily undergo magnetophoresis, facilitating drug diffusion throughout the tumor stroma; under a medium-frequency electromagnetic field (M*f*-EF), MNPs generate magnetothermal effects, enabling on-demand intracellular drug release *via* thermal responsiveness. Leveraging this principle, Liu *et al*. developed the DOX-PI/FVIOs system, in which doxorubicin is loaded onto FVIOs coated with the thermosensitive polymer PEI-IBAm 90. By sequentially switching the electromagnetic field frequency, the system achieves a cascade delivery process from tissue penetration to nuclear accumulation: under a 0.1 kHz low-frequency field, doxorubicin distributes uniformly and penetrates deeply within the tumor tissue (Figure 3A); subsequent exposure to a 360 kHz medium-frequency field significantly enhances doxorubicin fluorescence within cell nuclei (Figure 3B). In tumor-bearing mice, this strategy results in 86.2% of doxorubicin accumulating in tumor cell nuclei (Figure 3C), offering a novel approach to overcoming stromal barriers and improving targeted drug delivery efficiency.

**Figure 4:**
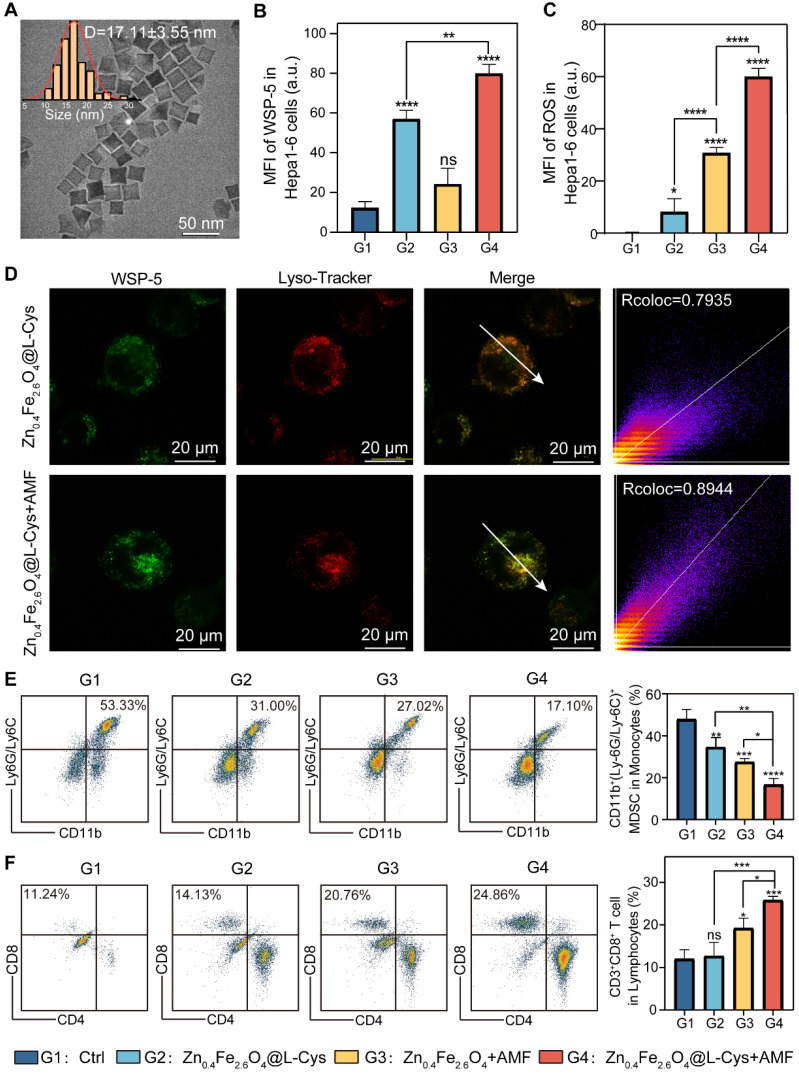
MH–assisted gas therapy for remodeling the tumor immune microenvironment. (A) Representative TEM image and corresponding size distribution of Zn_0.4_Fe_2.6_O_4_ nanoparticles. (B, C) Quantitative analysis of the mean fluorescence intensity of intracellular H_2_S and ROS in Hepa1-6 cells following different treatment conditions. (D) Colocalization analysis between the H_2_S-sensitive probe and lysosomes. (E, F) Percentages of myeloid-derived suppressor cells (MDSCs) and cytotoxic T lymphocytes in tumor tissues from mice subjected to different treatment regimens. Adapted with permission from 91. Copyright 2025, John Wiley & Sons, Inc.

With the progression of MNPs-based delivery systems, their design has shifted from simple drug loading and release toward multifunctional platforms capable of synergistic therapies. For example, Dai *et al*. developed a dual-responsive Zn_0.4_Fe_2.6_O_4_@L-Cys nanoplatform (Figure 4A) that simultaneously triggers L-cysteine release in the acidic lysosomal environment and its *in situ* conversion to H_2_S gas, while generating efficient MH under an AMF (Figure 4B–D) 91. By integrating “drug delivery–gas therapy–localized MH” into a single nanostructure, this platform enables precise localization and controllable release of therapeutic agents. It addresses the longstanding challenge of dose regulation in conventional H_2_S therapy and, at the same time, synergistically enhances ICD and alleviates the immunosuppressive TME, thereby markedly amplifying antitumor immune responses (Figure 4E, F).

In current cancer therapies, inefficient drug delivery remains a critical barrier to achieving optimal therapeutic outcomes. For instance, lenvatinib (LT), a first-line treatment for hepatocellular carcinoma, exhibits modest immunomodulatory effects as monotherapy but often fails to elicit robust antitumor immune responses, with dose-limiting toxicity further constraining its efficacy. To overcome these limitations, Ye *et al*. developed a polydopamine-coated, PEGylated FVIO@PDA-PEG nanocarrier for LT delivery 92. This platform responds to both the mildly acidic TME and MH, enabling dual pH- and heat-triggered drug release and establishing spatiotemporally confined chemothermal synergistic therapy within tumors. In hepatocellular carcinoma mouse models, this approach increased intratumoral cytotoxic T lymphocytes by ~3.86-fold, reduced regulatory T cell proportions to ~1.4%, and shifted cytokine profiles toward a proinflammatory, antitumor phenotype, ultimately achieving superior tumor suppression compared with monotherapy or hyperthermia alone. By integrating highly efficient FVIO-mediated MH with responsive drug release, this strategy forms a closed-loop, multifunctional system that combines localized hyperthermia, on-demand drug delivery, and TME remodeling, offering a promising avenue for enhancing antitumor immunity.

MNPs-based drug delivery systems guided by magnetic fields represent a novel strategy for tumor therapy. The main advantage of this approach is its capacity to target drugs directly to tumor tissues, enhancing local drug concentration while minimizing adverse effects on adjacent healthy tissues. However, MNPs-based drug delivery systems still encounter several challenges. Comprehensive evaluation of nanoparticle properties, biocompatibility, delivery efficiency, and *in vivo* metabolic pathways is required. Furthermore, precise control and modulation of magnetic fields demand advanced instrumentation and technical expertise. To achieve clinically viable magnetically controlled drug delivery, it is essential to develop nanocarriers that are both precisely controllable and inherently safe, while simultaneously advancing magnetic field generation and monitoring technologies capable of delivering stable, clinically permissible field strengths and frequencies.

## 5. MNPs-mediated MH induced antitumor immunity

Beyond its well-established application in local thermal ablation for glioblastoma and prostate cancer 93-95. MH has increasingly been recognized as a powerful modulator of systemic antitumor immunity. Growing evidence demonstrates that MH can contribute to systemic antitumor immunity, not only by induces local tumor regression via direct thermal cytotoxicity, but also by providing immunostimulatory cues that facilitate antigen presentation and effector activation.

MNPs-mediated heating and ROS generation can be considered upstream stressors that couple physical stimulation to immunological consequences through several interconnected steps. First, intracellular MH induces protein denaturation and membrane disruption, while ROS imposes oxidative damage to proteins, lipids, and nucleic acids 39. These stresses can converge on endoplasmic reticulum stress and unfolded protein response signaling, which are frequently associated with ICD. In this context, MH-treated tumor cells undergo a form of ICD that promotes the release of damage-associated molecular patterns (DAMPs), such as calreticulin (CRT) exposure on the cell surface, high-mobility group box 1 (HMGB1), and adenosine triphosphate (ATP). Together, these DAMPs provide a reasonable bridge from heat/ROS stress to enhanced immunostimulatory context. Second, these stress signals can influence antigen handling by antigen-presenting cells (APCs). DAMPs-driven uptake of dying tumor cells by DCs, may facilitate DC maturation and improve cross-presentation efficiency, thereby promoting antigen-specific T cell priming. These processes offer a mechanistic basis for the frequently reported linkage between MH and increased activation of antigen-specific cytotoxic T lymphocytes and natural killer (NK) cells. Third, MH may indirectly modulate immune infiltration and effector function via microenvironmental changes. MH can alter tumor perfusion and vascular permeability and may affect interstitial fluid pressure, which could influence immune cell infiltration into tumors. In addition, stress and damage responses can influence the levels of cytokines of chemokines, thereby impacting the composition and functional state of infiltrating immune populations. Collectively, this cascade of immune events supports the establishment of durable immune memory and may ultimately potentiate responses to immunotherapeutic interventions 96.

Importantly, the strength and direction of these immune effects are not uniform. They depend on MH parameters, nanoparticle properties, tumor context, and treatment time. Early studies by Kobayashi *et al*. also revealed that MH promotes CD4^+^ and CD8^+^ T cell infiltration into tumors, resulting in sustained and specific immune responses in rat models 97, 98. More recent studies further suggest that MH can synergize with immunotherapies by coordinating innate immune activation, ICD, and TME remodeling. In the following subsections, we summarize the key sections involved: innate immune, ICD, adaptive T-cell response, and combination with immune checkpoint blockade.

### 5.1 MH induces efficient ICD

ICD is considered a regulated form of cell death that actively elicits adaptive immune responses, and is characterized by a sequence of spatiotemporally controlled DAMPs, such as the exposure CRT on the tumor cell surface and the release of extracellular ATP and HMGB1. These signals can be recognized and internalized by DCs and other APCs, thereby promoting antigen cross-presentation and the activation of CD8^+^ T cells. Compared with conventional hyperthermia, MH features nanoscale, intracellularly confined heating, which is frequently accompanied by pronounced ROS generation and sublethal cellular stress. This state is more likely to induce ICD rather than nonspecific necrotic damage 99-101.

**Figure 5:**
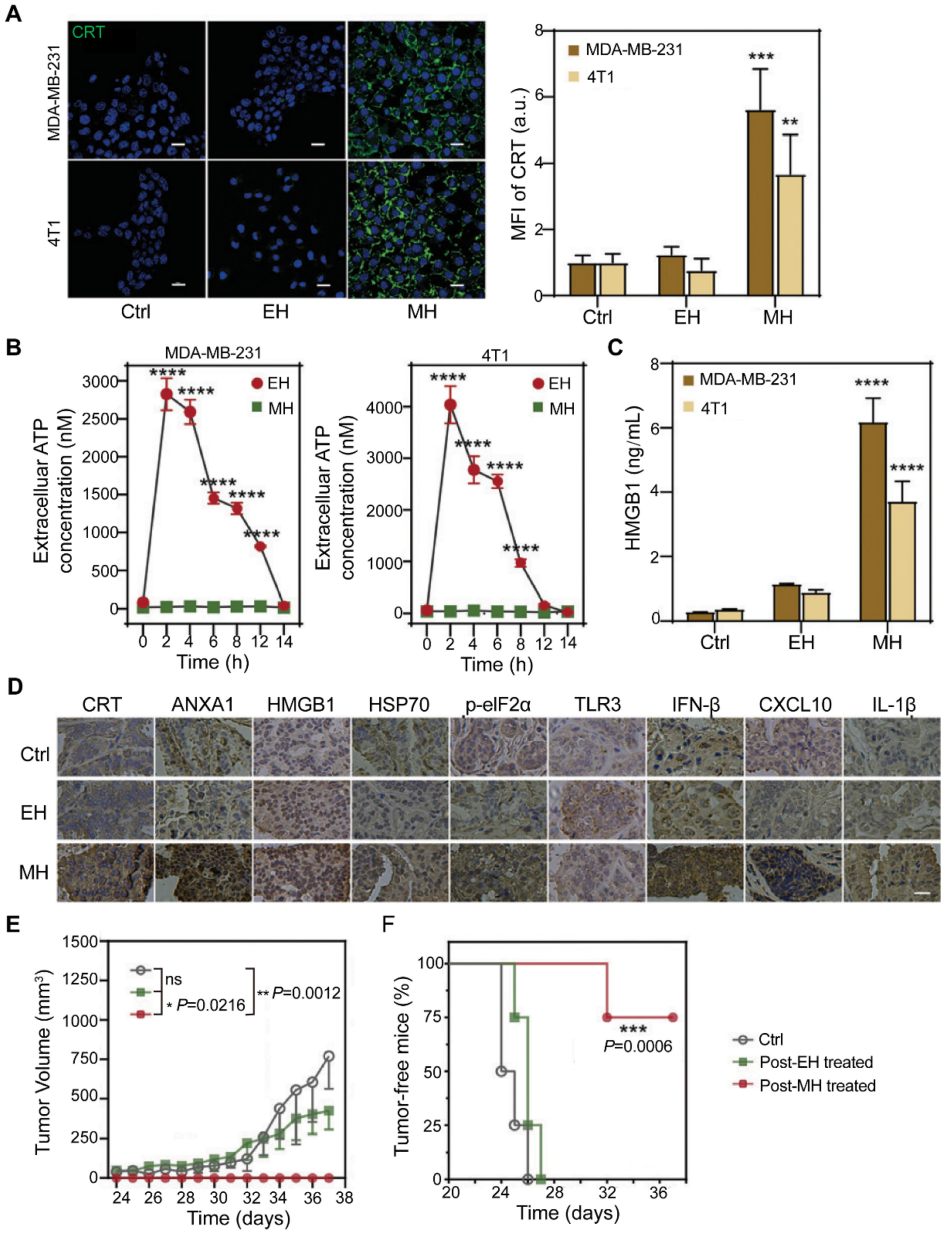
MH–induced ICD. (A) Confocal fluorescence images and quantification of CRT exposure on cancer cells following treatment. (B) Extracellular ATP release from cancer cells at various time points post-treatment. (C) HMGB1 release quantified by ELISA. (D) Immunohistochemical visualization of DAMPs in tumor tissues *ex vivo*. (E) Tumor growth curves of mice subjected to different treatment regimens. (F) Percentage of tumor-free surviving mice. Adapted with permission from 102. Copyright 2022, Royal Society of Chemistry.

Yan *et al.* provided direct evidence that MH can elicit bona fide ICD in breast cancer models 102. MH treatment robustly induced the surface exposure of CRT on cancer cells (Figure 5A) and promoted the extracellular release of ATP and HMGB1 (Figure 5B, C). In addition, MH enhanced the secretion of immunostimulatory factors, including HSP70/90 and type I interferons, in patient-derived tumor tissues (Figure 5D). Crucially, tumor cells killed by MH conferred protective immunity against subsequent tumor challenges (Figure 5E, F), confirming that MH-induced cell death is functionally immunogenic and capable of triggering systemic antitumor immune responses.

**Figure 6:**
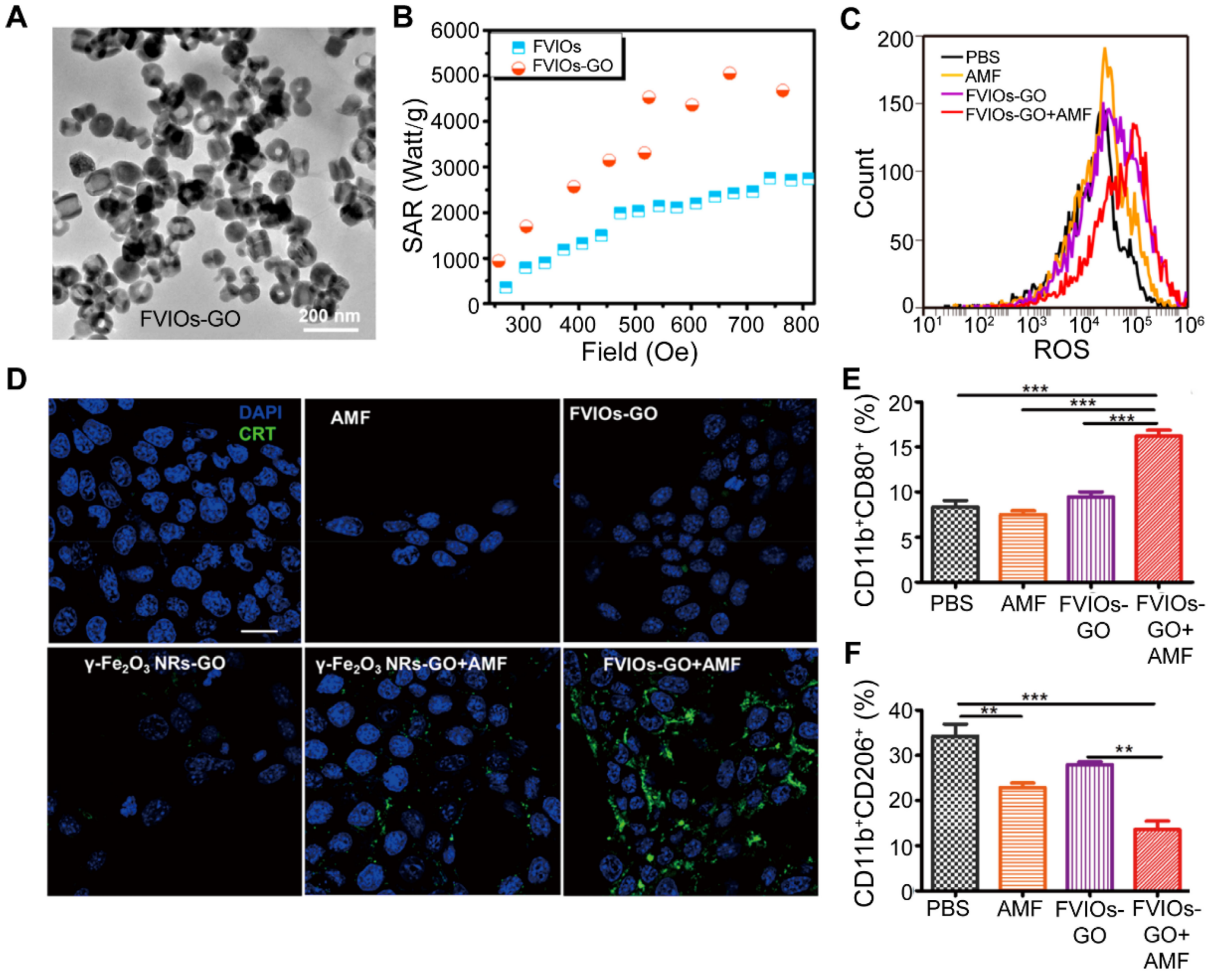
FVIOs-GO–mediated MTD induces ICD and promotes M1 macrophage polarization. (A) TEM image of FVIOs-GO. (B) Comparison of SAR values between FVIOs-GO and FVIOs under different magnetic field strengths. (C) Flow cytometric analysis of intracellular ROS levels in 4T1 cells following various treatments. (D) Confocal images showing CRT exposure on the surface of 4T1 cells after different treatments. (E, F) Quantitative analysis of M1 and M2 macrophage populations in the tumor microenvironment after MTD. Adapted with permission from 82. Copyright 2020, American Chemical Society.

To further enhance this effect, the same research group developed a magnetothermodynamic (MTD) therapy 82, which integrates a high SAR with augmented ROS generation (Figure 6A-C). This synergistic strategy induced significantly stronger ICD markers compared with conventional MH (Figure 6D) and facilitated the repolarization of TAMs from the pro-tumorigenic M2 phenotype to the antitumorigenic M1 phenotype (Figure 6E, F), thereby achieving superior *in vivo* antitumor efficacy at relatively low doses.

The subcellular localization of heat delivery can further enhance the induction of ICD. Zhang *et al.* demonstrated that lysosome-targeted MNPs mediating lysosomal MH activate specific apoptotic pathways and caspase-1–dependent cytokine secretion, thereby amplifying CRT exposure and overall ICD efficiency 103. These results highlight that the intracellular fate of MNPs can be precisely engineered to optimize immunogenic responses.

These ICD-related events provide the critical upstream signals for efficient antigen cross-presentation and the priming of effector T cells, as DAMPs and inflammatory cytokines drive DCs recruitment, maturation, and migration to draining lymph nodes (dLNs). In this context, tumor-associated antigens (TAAs) derived from MH-treated cells are more efficiently processed and presented *via* MHC pathways, supporting robust activation, clonal expansion, and functional differentiation of CD8^+^ cytotoxic T lymphocytes and CD4^+^ helper T cells, which together underpin durable, systemic antitumor immunity. Notably, ICD provides a set of integrated antigenic and adjuvant signals that facilitates DC maturation and migration to draining lymph nodes. After this priming phase, the key determinant of therapeutic outcome is whether sufficient numbers of functional tumor-specific T cells can be generated and effectively infiltrate the tumor site.

### 5.2 MH and innate immunity

**Figure 7:**
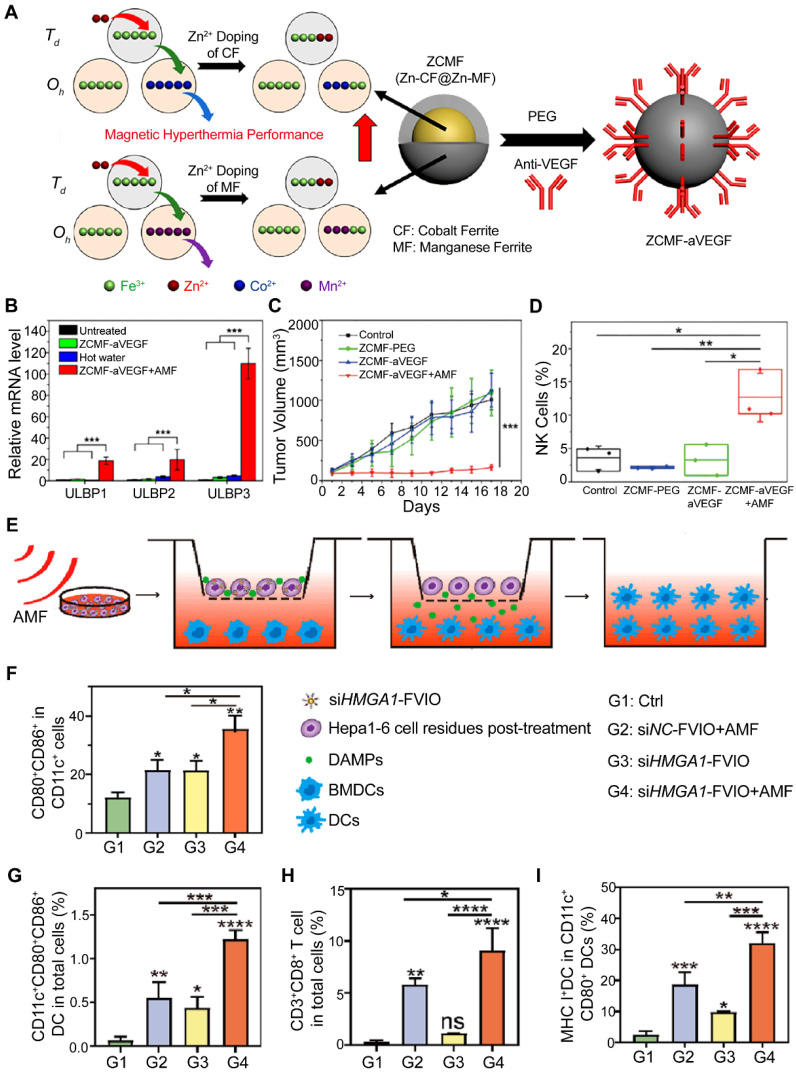
Activation of innate antitumor immunity by MH. (A) Schematic illustration of the magnetically enhanced core–shell nanohybrid ZCMF-αVEGF. (B) Effects of different treatments on the mRNA expression of NK cell–activating ligands in HepG2 cells. (C) Tumor growth curves of tumor-bearing mice under different treatment regimens. (D) Quantification of tumor-infiltrating NK cells in tumor tissues. Adapted with permission from 39. Copyright 2021, John Wiley & Sons, Inc. (E, F) Effects of different treatments on the expression of DCs maturation surface markers in Transwell assays conducted *in vitro*. (G–I) Flow cytometric analysis of mature DCs, infiltrating CD8^+^ T cells, and MHC I^+^ DCs in Hepa1-6 tumor tissues. Adapted with permission from 105. Copyright 2023, American Chemical Society.

Innate immunity represents the first line of defense against tumor initiation and progression, and innate immune cells are typically the earliest responders to stress signals from dying cancer cells. However, its antitumor activity is frequently suppressed within the TME. By locally remodeling the TME, MH has emerged as an effective approach to overcome this immunosuppressive state and restore innate immune function. In particular, MH can reactivate key innate immune effector cells, including NK cells, DCs, and macrophages 104. Pan *et al.* developed an exchange-coupled core–shell nanohybrid (ZCMF-αVEGF) for MH treatment (Figure 7A) 39. This nanoplatform not only inhibited hepatocellular carcinoma growth but, more importantly, enhanced NK cell–mediated antitumor immunity by upregulating stress-associated ligands on tumor cells (Figure 7B). As a result, both primary and metastatic tumor growth were effectively suppressed *in vivo* (Figure 7C, D), underscoring that the therapeutic efficacy of MH extends beyond direct thermal ablation and involves active immune reprogramming of the TME.

DCs are pivotal for initiating adaptive immune responses 106, 107. Among APCs, DCs play a key role in initiating and regulating both innate and adaptive immune responses 108-111. Tumor cell debris generated by MH has been demonstrated to serve as a potent source of tumor antigens, effectively promoting DCs maturation *in vitro* 37. These findings suggest that MH can act as *in situ* vaccination strategy to enhance antigen presentation. Expanding on this approach 112. Yan *et al.* utilized FVIOs to deliver siRNA targeting HMGA1, an immunosuppressive protein overexpressed in hepatocellular carcinoma (Figure 7E, F) 105. In murine models, the combined application of HMGA1 silencing and MH substantially enhanced DCs maturation and MHC-I–mediated antigen presentation, leading to pronounced infiltration of DCs and CD8^+^ T cells into the TME (Figure 7G-I).

Tumor-associated macrophages (TAMs) constitute a pivotal component of the innate immune system 113, 114. Jiang *et al.* demonstrated that factors released from MH-treated tumor cells can recruit and activate macrophages, enhancing their phagocytic activity *via* regulation of the RAB7 and SIRPα signaling pathways 115. Expanding on these findings, Wang *et al.* first revealed that FVIO-mediated MH concurrently suppresses the “don’t eat me” signal (CD47/SIRPα) on tumor cells while promoting the “eat me” signal (CRT), effectively nearly doubling macrophage-mediated phagocytosis 116. Collectively, these studies highlight that MH can powerfully activate multiple innate immune effectors—including NK cells, DCs, and macrophages—reprogramming the TME from an immunosuppressive to an immunostimulatory state and providing a solid mechanistic basis for combining MH with immunotherapy. Notably, many of the innate changes above are initiated by signals released from MH-stressed tumor cells.

### 5.3 MH enhances T cell-mediated antitumor response

T cells play a pivotal role in eliminating cancer cells and account for the majority of adaptive antitumor immune responses 117. However, due to the dense stromal architecture of solid tumor, limited intrinsic immunogenicity, and multiple immunosuppressive mechanisms within the TME. T cells often struggle to efficiently infiltrate tumor tissue or to maintain sustained cytotoxic activity once they arrive, ultimately leading to immune escape 118. Therefore, enhancing T-cell infiltration into the tumor core and restoring T-cell function within the TME have become critical strategies to improve immunotherapy efficacy.

**Figure 8:**
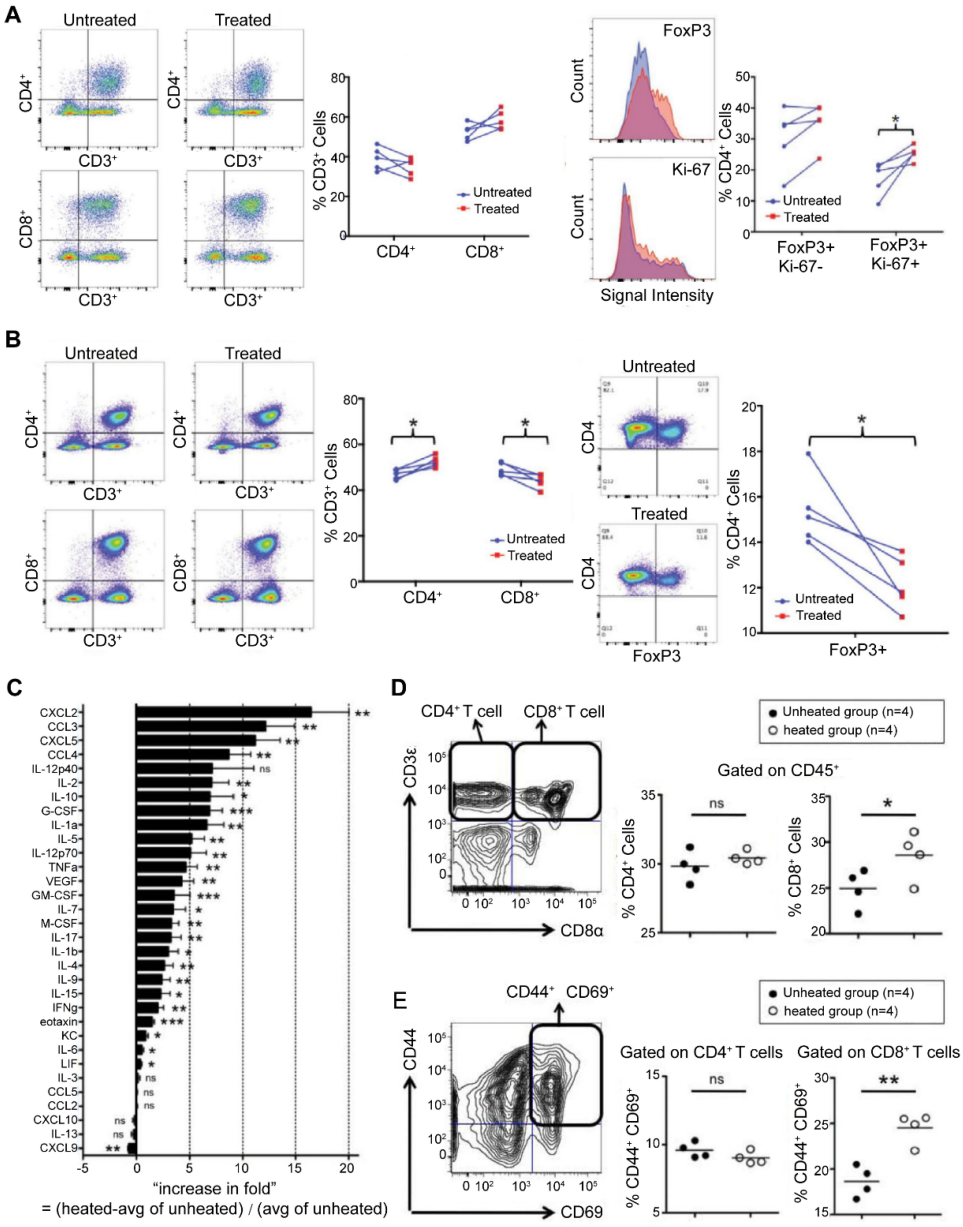
MH promotes T cell infiltration and activation. (A) Flow cytometric analysis of intratumoral T cell subsets, including proliferating and regulatory T cells. (B) Flow cytometric analysis of T cell subsets and regulatory T cells in dLNs. Adapted with permission from 119. Copyright 2021, John Wiley & Sons, Inc. (C) Alterations in cytokine and chemokine levels within tumors following MH treatment. (D) Proportions of CD4^+^ and CD8^+^ T cells among leukocytes in dLNs. (E) Proportions of activated (CD44^+^CD69^+^) CD4^+^ and CD8^+^ T cells in dLNs. Adapted with permission from 120. Copyright 2014, Elsevier Inc.

MH has emerged as a promising strategy to enhance antitumor immunity. In glioma models, Carter *et al.* demonstrated that MH treatment not only inhibited tumor growth but also promoted the infiltration of CD4^+^ and CD8^+^ T cells within the tumor and the dLNs 119. These results indicate that MH can effectively convert immunologically “cold” tumors into “hot” tumors with increased lymphocyte infiltration, thereby creating a more immunologically responsive microenvironment (Figure 8A, B).

Complementing these observations, Toraya Brown *et al.* provided mechanistic insights in a melanoma model 120. They demonstrated that MH elicited broad upregulation of proinflammatory cytokines and chemokines within the TME (Figure 8C). This immunologically remodeled TME was accompanied by a significant increase in both the proportion and activation status (CD44^+^CD69^+^) of tumor-specific CD8^+^ T cells in the dLNs (Figure 8D, E), correlating with protective immunity against subsequent tumor rechallenge. Together, these findings establish MH as a potent bridge between localized hyperthermia and systemic T cell–mediated antitumor immunity. By inducing ICD and reshaping the cytokine landscape of the TME, MH promotes the recruitment, activation, and sustained effector function of tumor-specific T cells, thereby overcoming a key bottleneck in current immunotherapeutic approaches. However, even when MH increases T-cell priming and infiltration, T cells can still be restrained by checkpoint pathways and suppressive myeloid programs in the TME. This limitation provides a direct rationale for pairing MH with immune checkpoint blockade.

### 5.4 Combined therapy of MH and ICB

**Figure 9:**
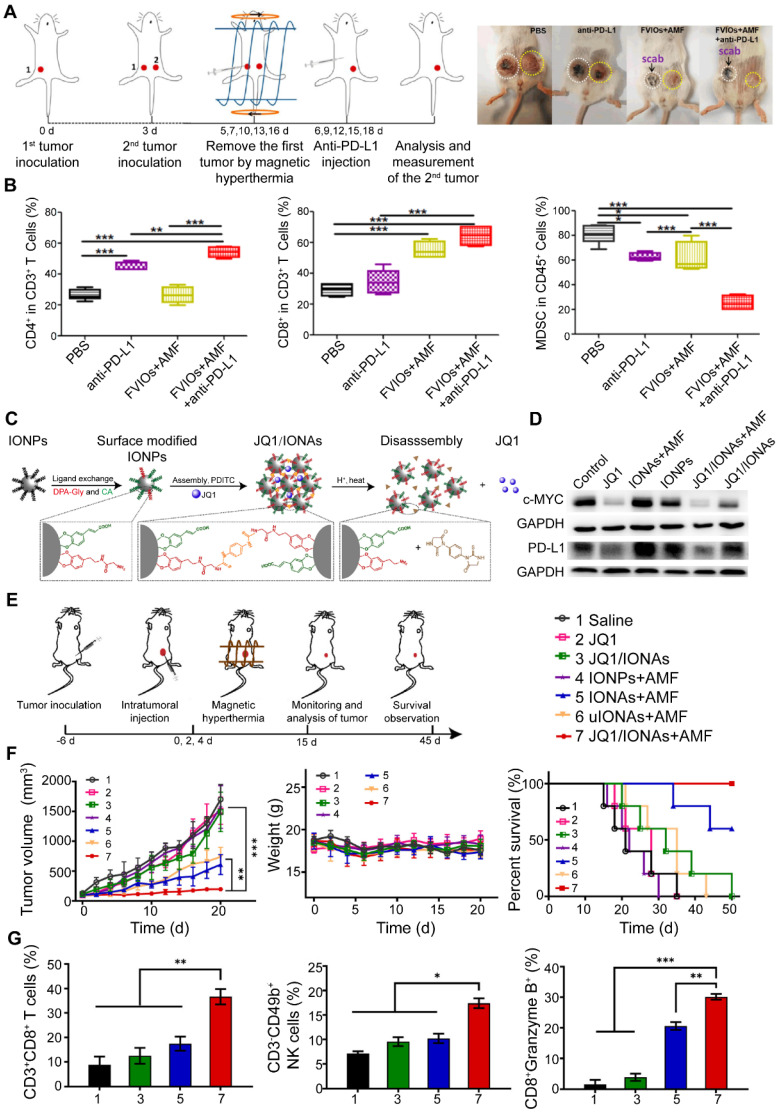
Enhancement of antitumor immune responses by MH combined with PD-1/PD-L1 blockade. (A) Experimental design for MH in combination with anti–PD-L1 therapy, with representative images of treated mice. (B) Immune activation induced by the combination therapy, demonstrated by increased CD4^+^ and CD8^+^ T cell infiltration and reduced MDSCs in distant tumors. Adapted with permission from 36. Copyright 2019, American Chemical Society. (C) Schematic illustration depicting the preparation and disassembly of thermosensitive iron oxide nanostructures loaded with JQ1. (D) Western blot analysis of c-MYC and PD-L1 expression following various treatments. (E) Schematic overview of the *in-situ* tumor model establishment and treatment protocol. (F) Tumor growth curves, body weight changes, and survival curves of mice under different treatment regimens. (G) Analysis of intratumoral immune responses, including proportions of CD8^+^ T cells, NK cells, and granzyme B levels, following different treatments. Adapted with permission from 127. Copyright 2023, Elsevier Inc.

Given that many solid tumors exhibit primary or acquired resistance to immune checkpoint blockade (ICB), there is a clear need for rational combination strategies that can convert limited immune activation into durable antitumor immunity. In this context, MH provides a mechanistically grounded partner for ICB by inducing ICD and remodeling the TME to enhance antigen presentation and effector immune function. ICB therapy, which targets inhibitory receptors such as PD-1 on T cells or PD-L1 on tumor cells, has fundamentally transformed cancer treatment 121-124. Nevertheless, its efficacy in many solid tumors is often limited by primary or acquired resistance 125, prompting the investigation of effective combination strategies. MH, through the induction of ICD and remodeling of the TME, provides a rational approach to sensitize tumors to ICB. Liu *et al.* demonstrated this synergistic effect using FVIOs–mediated MH combined with anti–PD-L1 therapy 36. This combination not only increased the proportion of cytotoxic T lymphocytes but also reduced the prevalence of immunosuppressive myeloid-derived suppressor cells within the TME. Functionally, it achieved superior control of both primary and metastatic breast tumor growth (Figure 9A, B), highlighting how MH can enhance the therapeutic index of checkpoint inhibition.

Beyond antibody-mediated PD-1/PD-L1 blockade, direct suppression of PD-L1 expression in tumor cells represents a complementary strategy. Gao *et al.* developed a thermosensitive iron oxide nanocomposite co-loaded with a BET inhibitor capable of downregulating PD-L1 transcription (Figure 9C) 126. MH triggered site-specific drug release, effectively reducing PD-L1 expression *in vivo* and leading to complete tumor regression (Figure 9D–F). This approach also promoted enhanced infiltration of CD8^+^ T cells and NK cells, along with increased granzyme B production (Figure 9G), thereby eliciting a robust and coordinated antitumor immune response.

The immunomodulatory effects triggered by MH represent a burgeoning field that brings new prospects and opportunities to cancer treatment. Leveraging the local MH effect within tumors, it offers a precise regulatory method to improve the tumor immune microenvironment. Unlike conventional bulk hyperthermia, which primarily acts at the tissue level through direct thermal ablation, MH operates at the cellular and subcellular scales, delivering localized intracellular heating that not only compromises tumor cell viability but also induces ICD, promotes DAMPs release, reshapes cytokine and chemokine profiles, and modulates key checkpoint pathways. As a result, MH can transform the tumor site from a relatively immunologically “silent” state into an immune-active state that supports T-cell priming, effector function, and long-term immune memory. Importantly, the immunological consequences of MH extend beyond simple tumor cell destruction and play a decisive role in enhancing antigen presentation, recruiting and activating immune effector cells, and preventing tumor relapse. Recent studies further indicate that tumor cells undergoing MH-triggered ICD can serve as a rich source of TAAs and DAMPs, making them attractive candidates for whole-cell vaccine development. This concept positions MH as a versatile technique not only for direct local tumor killing but also for *in situ* vaccine generation and rational combination with ICB, tumor vaccines, and other immunotherapeutic modalities. Such integrative approaches may ultimately broaden the population of patients who benefit from immunotherapy and improve the depth and durability of clinical responses.

## 6. MNPs-mediated nanovaccines for tumor immunotherapy

Against this backdrop of MH-induced ICD, CTL activation, and synergistic effects with ICB, it becomes clear that initiating local immunity is only the first step toward a systemic antitumor response. A major challenge in current cancer immunotherapy is how to further amplify this localized immune activation into a durable, body-wide defense capable of controlling metastasis and preventing recurrence. To this end, therapeutic cancer vaccines represent a more advanced immunotherapeutic strategy 127, 128. They are designed to convert tumor-derived TAAs into effective antigens. Through rational adjuvant design and dLNs targeting, cancer vaccines aim to enhance antigen-specific T cell responses and establish long-term immune memory 129, 130. It should be noted that direct evidence for MH-enabled tumor vaccine preparation is still lacking. However, mechanistic insights from MH studies–especially the generation of tumor antigens and danger signals, as well as the critical requirement for efficient antigen delivery and DCs programming–provide a clear conceptual basis for the rational design of engineered vaccine platforms. Despite these theoretical advantages, cancer vaccines continue to face substantial clinical challenges, including inefficient and slow delivery as well as limited activation of adaptive immune responses. Consequently, their therapeutic potential has yet to be fully realized 131, 132. In recent years, MNPs have been widely explored as modular vaccine scaffolds and delivery carriers due to their good biocompatibility, tunable surface chemistry, and magnetic field-assisted controllability. These features offer new opportunities to overcome the limitations of conventional cancer vaccines by improving antigen delivery efficiency and enhancing immune activation.

**Figure 10:**
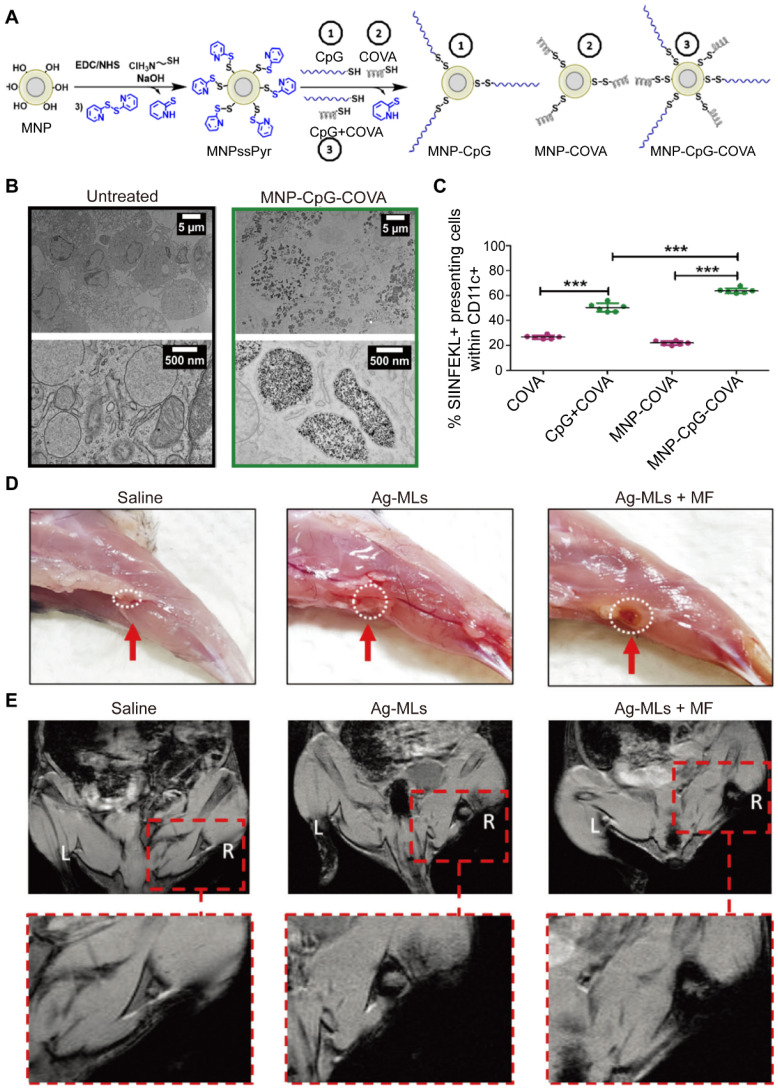
Magnetic field–guided tumor vaccines enhance antigen uptake and DCs maturation. (A) Schematic representation of MNPs covalently conjugated with CpG adjuvant and OVA antigen. (B) TEM images of bone marrow–derived dendritic cells (BMDCs) before and after internalization of MNP-CpG-OVA. (C) Percentage of CD11c^+^ BMDCs presenting MHC I–OVA complexes following co-incubation with different formulations. Adapted with permission from 134. Copyright 2023, Elsevier Ltd. (D) *In vivo* anatomical imaging showing Ag-MLs accumulation in lymph nodes under magnetic targeting. (E) T2-weighted magnetic resonance imaging illustrating enhanced delivery of Ag-MLs to targeted lymph nodes. Adapted with permission from 130. Copyright 2023, John Wiley & Sons, Inc.

MNPs are particularly attractive as core components of nanovaccine platforms due to their favorable biocompatibility, physicochemical stability, and highly versatile surface functionalization. For example, Nuria *et al.* developed a multifunctional magnetic nanoparticle, MNP-CpG-COVA, composed of a γ-Fe_2_O_3_ core surface-functionalized with the antigenic peptide COVA and the immune adjuvant CpG (Figure 10A) 133. Importantly, both COVA and CpG were covalently linked to the magnetic core through disulfide bonds, which substantially improved the stability of the construct in systemic circulation. Exploiting the intracellular–extracellular gradient of glutathione, MNP-CpG-COVA was designed to release COVA and CpG specifically after cellular uptake (Figure 10B). This strategy not only enhanced DCs activation efficiency but also significantly improved their antigen-presenting capacity (Figure 10C), thereby providing a more favorable platform for subsequent T cell priming and expansion.

Magnetic field–guided delivery represents one of the major advantages of MNPs. Sheng *et al.* encapsulated soluble murine melanoma lysates as TAAs together with SPIOs into a liposomal carriers, and further functionalizing the liposome surface with the immune adjuvant CpG-1826 to generate antigen-loaded magnetic liposomes (Ag-MLs) 129.* In vitro*, Ag-ML was efficiently internalized by bone marrow–derived DCs, leading to robust DCs activation and subsequent T cell proliferation. *In vivo* studies in a murine melanoma model showed that, compared with passive administration, application of a 0.5 T magnetic field to the popliteal region significantly enhanced the accumulation of Ag-ML in tumor-dLNs (Figure 10D, E). This magnetic targeting significantly increased DCs activation and expanded the population of antigen-specific CD8^+^ T cells in both the tumor and spleen, ultimately improving overall survival in treated mice. Furthermore, by incorporating a fluorescent probe (multifunctional indocyanine green), the biodistribution and lymph node accumulation of Ag-MLs could be directly visualized, further confirming their magnetic responsiveness. Collectively, these findings highlight that magnetic field–assisted delivery, coupled with surface modification or pre-loading into DCs, can substantially improve lymph node targeting, strengthen DCs antigen presentation, and enhance T cell priming and activation 134. This body of work provides a strong experimental foundation for the rational integration of magnetic nanomaterials with cancer vaccine strategies and other immunotherapies in future translational applications.

Beyond serving as standalone vaccine carriers, MNPs can be leveraged to engineer functionally enhanced DCs–based vaccines. Huang *et al*. developed Fe_3_O_4_@Ca/MnCO_3_ core–shell nanoparticles capable of loading the model antigen ovalbumin (OVA) 135. Under magnetic guidance, these nanoparticles were efficiently internalized by DCs and subsequently degraded within lysosomes, resulting in the concomitant release of OVA and Mn^2+^/Ca^2+^ ions. While the released antigen provides epitopes for antigen presentation, the liberated metal ions—well-recognized modulators of innate immune signaling—are thought to supply additional stimulatory cues that promote DCs maturation. Consistent with this mechanism, co-culture experiments demonstrated enhanced priming and expansion of antigen-specific cytotoxic T lymphocytes. *In vivo*, DCs preloaded with these nanoparticles exhibited improved homing to dLNs. As a complementary approach, Chen *et al*. engineered an iron oxide nanoparticle–based nano-adjuvant for the co-delivery of a stimulator of interferon genes (STING) agonist and antigen 136. This platform displayed pH-responsive disassembly, facilitating endosomal/lysosomal escape and targeted delivery to antigen-presenting cells within lymph nodes. Consequently, it elicited robust and durable CD8^+^ T cell responses, effectively suppressing tumor growth and metastasis, and exhibited pronounced synergy with anti–PD-L1 immunotherapy.

Overall, the integration of MNPs into cancer vaccine design offers substantial advantages. Magnetic guidance and tunable surface functionalization can improve the delivery of antigens and adjuvants dLNs, while controlled ion release and MH or ICD-related mechanisms can amplify both innate and adaptive immune responses. Nevertheless, future studies are needed to define the structure and response relationships of magnetic nanovaccines in a more quantitative manner, including the impact of particle size, sharp, composition, and magnetic properties, as well as dosing regimens, on vaccine performance. Equally important is a comprehensive understanding of their behavior under clinically relevant magnetic field conditions (field strength, frequency, exposure schedule) and their long-term biosafety, degradation, and clearance profiles. These efforts will be essential to improve the precision, efficiency, and controllability of magnetic vaccine strategies, thereby enabling more reliable and effective application of these platforms in cancer immunotherapy.

## 7. Limitations and advantages of MH in clinical transformation

### 7.1 Current status of clinical translation of MH

NanoTherm^®^, the world's first magnetic hyperthermia system approved for tumor treatment, has demonstrated significant survival benefits in the clinical application for recurrent glioblastoma, and the exploration of combination therapies has further expanded its potential and application boundaries. In 2011, MAGFORCE AG, a German company, published Phase I/II clinical trial data on the treatment of brain gliomas with this system 137. In this study, 50 glioma patients were injected with magnetic nanoparticles (MNPs) intraoperatively, followed by six sessions of NanoTherm^®^ magnetic hyperthermia (60 minutes per session) combined with radiotherapy. The results showed a median survival of 13.4 months for patients, significantly better than the radiotherapy-only group (6-9 months), with an objective response rate as high as 40%. In 2021, a clinical trial for NanoTherm^®^ in the treatment of prostate cancer (NCT05010759) was launched in the United States, and the data showed a significant decrease in prostate-specific antigen (PSA) levels in 90% of the enrolled patients, providing preliminary validation of its value in this indication. However, NanoTherm^®^ still faces significant technical limitations. Due to the inherent performance shortcomings of the magnetic hyperthermic agents, its heat generation efficiency is limited under the clinically used magnetic field strengths (100-300 kHz). Using higher magnetic field strengths to enhance the heating effect leads to adverse reactions such as groin pain and skin burning sensations. More critically, in previous clinical trials, all patients treated with NanoTherm^®^ developed peritumoral edema, 40%-67% experienced neurological deterioration, and 3% of patients required a second surgery to remove residual nanoparticles and surrounding proliferative granulation tissue due to refractory edema.

Although the clinical performance of NanoTherm^®^ pioneering exploration in the field of magnetic hyperthermia has laid an important clinical foundation and practical basis for subsequent technological development. However, their widespread application remains constrained by a series of interrelated, fundamental challenges spanning from production to bedside implementation. These challenges not only concern the technology itself but also highlight the core bottlenecks in translating complex nanosystems into routine clinical therapies. First, there is an inherent conflict between the scalability of production and the uniformity control across batches. While laboratory-scale synthesis allows precise control, maintaining this nanoscale uniformity during large-scale (GMP-grade) clinical production is extremely difficult. Even minor batch-to-batch variations can be amplified, ultimately resulting in unpredictable heat dose delivery.

Second, systematic evaluation of long-term biocompatibility and degradation-clearance pathways remains insufficient. Although the iron oxide core is generally considered to be metabolized through the human iron pathway, its long-term fate *in vivo*, particularly after surface functionalization and accumulation at tumor sites, remains largely unknown. Data on the gradual degradation kinetics of nanoparticles under physiological conditions, the chemical forms of their degradation products, and potential retention effects in sensitive tissues such as the brain are lacking over multi-year timeframes.

More prominently, the complexity of the therapeutic procedure and technical accessibility constitutes a physical barrier to clinical adoption. Current protocols rely on two consecutive, highly specialized steps: precise delivery of MNPs to the lesion site and MH treatment using dedicated alternating magnetic field devices. This strong dependence on invasive surgical procedures and large specialized equipment restricts application to a limited number of medical centers. Therefore, broad clinical implementation requires both procedural and device optimization as well as the establishment of standardized expert consensus guidelines. Additionally, patient-specific factors—such as individual metabolism, blood flow rates, and tumor location—necessitate personalized treatment plans. The rapid development of artificial intelligence offers promising prospects for enabling such individualized therapy.

### 7.2 Limitations and current challenges of MNPs-mediated MH

Despite encouraging progress, the clinical translation of magnetic nanoparticle-mediated MH remains constrained by four major core challenges, which involve the precise delivery of MH agents, their distribution in the body, the impact of physiological environments, and potential immune-related side effects. Addressing these issues, an increasing number of studies have proposed solutions.

Firstly, regarding the delivery of MH agents, nanoparticles often struggle to achieve uniform distribution within tumor tissues. The dense extracellular matrix and high interstitial fluid pressure in tumors hinder the diffusion of particles, often leading to heat concentration near the injection site and creating treatment blind spots in distal areas. To address this, functional coatings, such as hyaluronidase, have been developed to degrade the matrix barrier, combined with image-guided multi-point injection techniques to optimize spatial distribution.

Secondly, the heat sink effect, where blood flow inside or around the tumor rapidly carries away heat, is a key obstacle affecting the heating efficiency. Areas near large blood vessels struggle to reach effective temperatures. To overcome this issue, arterial embolization can be applied in clinical practice to preemptively block blood supply to the lesion area. This not only counteracts the cooling effect of blood perfusion but also maximizes the retention of MNPs in the lesion region.

Thirdly, the phagocytosis of nanoparticles by macrophages significantly limits tumor accumulation. Most intravenously injected particles are engulfed by macrophages in the liver and spleen, with less than 5% of the dose reaching the target area. To address this, “stealth” coatings, such as polyethylene glycol, are used to modify the surface of the particles, reducing serum protein adsorption and extending circulation time. Additionally, direct intratumoral injection is the most straightforward method to bypass this clearance mechanism.

Finally, in clinical scenarios requiring multiple treatments, repeated administration or stimulation may trigger immune-related adverse reactions. To resolve this, cellular-derived membrane structures are used to camouflage MNPs, or immunomodulatory agents are used when necessary to temporarily suppress the activation of the complement system.

In summary, the clinical application of MH is gradually overcoming these physical and physiological barriers through material innovations and optimized combination treatment strategies.

### 7.3 Advantages of MH in cancer treatment

MH exhibits significant differences in mechanism, therapeutic advantages and limitations, and immune effects compared to commonly used clinical thermal therapies such as photothermal therapy (PTT), radiofrequency ablation (RFA), and microwave hyperthermia. Understanding these differences helps to highlight the unique therapeutic value of MH.

The core advantage of MH lies in its extremely high spatial and temporal control precision. Nanoscopic heating allows for the precise destruction of target cells without harming surrounding healthy tissues. Moreover, it allows for repeated treatments and has no penetration depth limitations, relying only on the magnetic field for tissue penetration, with minimal side effects. In contrast, PTT is limited by the shallow tissue penetration depth of light (typically < 10 mm), and is easily affected by skin pigmentation and tissue scattering, making it suitable primarily for superficial tumors 138, 139. Furthermore, high-temperature-induced heating may damage adjacent normal tissues. RFA is known for its high treatment efficiency and precise ablation of medium to superficial solid tumors, with mature clinical applications. However, it has poor control over the heating range, leading to the “heat sink effect” (such as difficulty reaching effective temperatures in tissues near blood vessels), and is invasive, often resulting in postoperative complications such as pain and bleeding, with limited potential for repeated treatments 140. Microwave ablation offers intermediate tissue penetration depth and can heat larger areas of tissue, making it advantageous for diffuse lesions 141. However, it suffers from poor spatial control precision and uneven heating, which can lead to thermal damage to normal tissues, and its therapeutic effect is highly dependent on tissue water content.

Importantly, unlike whole-body hyperthermia, intracellular magnetic hyperthermia uniquely enables organelle-specific stress, controllable immunogenic cell death without necrosis, and direct modulation of immune cell function. These features are not achievable with conventional thermal therapies. MH, through intracellular nanoscopic gentle heating (usually 41-43°C), can precisely induce ICD in target cells, efficiently releasing DAMPs, while avoiding the inflammatory storm caused by large-scale tissue necrosis. This significantly promotes the maturation of APCs, enhances the infiltration of effector T cells, and effectively reverses the tumor immune-suppressive microenvironment, even inducing systemic antitumor immune memory, which reduces tumor recurrence and metastasis. In contrast, PTT, RFA, and microwave hyperthermia primarily rely on high-temperature ablation, which can cause large-scale tissue necrosis and trigger intense local inflammatory responses. However, these inflammatory responses lack specificity and are prone to causing damage to normal tissues. Moreover, immune cell infiltration in the ablation area is insufficient, making it difficult to induce a systemic immune response. In fact, tissue necrosis fragments may even lead to immune suppression, preventing the formation of long-lasting antitumor immunity.

### 7.4 Image-mediated MH can dynamically monitor the treatment process

MH is intrinsically suited for a theranostic paradigm because magnetic nanoparticles can serve simultaneously as heat mediators and imaging probes. Magnetic resonance imaging (MRI) enables visualization of nanoparticle accumulation and provides indirect thermal information through MR thermometry 34, 142, 143. Magnetic particle imaging (MPI) allows quantitative mapping of nanoparticle distribution without tissue background, enabling estimation of relative delivered dose 144, 145. Photoacoustic imaging can report optical absorption and temperature-related changes in tumor tissues, whereas PET/SPECT labeling of magnetic nanoparticles provides sensitive whole-body biodistribution and pharmacokinetic information. Importantly, these imaging readouts can be directly linked to immunotherapeutic decision-making, including adjustment of AMF parameters to achieve immunogenic heating thresholds, optimization of nanoparticle dosing for efficient antigen release, and spatial targeting of heating to promote local immune activation while limiting systemic inflammation. When combined with temperature monitoring and adaptive field modulation, such strategies establish a basis for feedback-controlled MH that coordinates thermal dosing with immune modulation, thereby improving both antitumor immunity and treatment safety.

## 8. Conclusion and perspective

There are numerous advantages to leveraging MH in anti-cancer treatment, such as high selectivity, precision, and low toxicity to healthy cells. As the significance of hyperthermia in cancer therapy gains recognition, research efforts should now intensify to harness the great potential of MNPs-mediated MH in boosting anti-tumor immunity.

Multiple challenges persist in MH treatment, as exemplified by NanoTherm^®^ (comprising amino silane-coated Fe_3_O_4_ nanoparticles) being the sole magnetic nanoagent (at the time of writing) approved for patients undergoing MH treatment. However, due to their low magnetic heating efficiency, they are unable to meet more stringent clinical requirements. Some other MH agents are currently in the development stage, and more positive results need to be observed before their clinical application. Evidence suggests that intracellular ROS have a crucial role in cell signaling and function regulation, emphasizing the need to optimize the physical parameters (*e.g.*, volume, composition, and morphology) of MNPs to enhance their magnetic responsiveness and Fenton catalytic activity, thereby increasing ROS generation. However, the potential release of bioactive free iron from MNPs poses safety risks, raising concerns about the toxicity and degradation of normal tissue during clinical application. Additionally, due to the accumulation of immunotherapeutic drugs in the body, which may induce autoimmune diseases and immune-related adverse reactions, it is imperative to carefully consider efficacy, bioavailability, and off-target effects when designing MNPs-based delivery systems for hydrophobic immunotherapeutic agents. To better combine magnetic field and hyperthermia effects, the relationship of AMF parameters and magnetic responsive behaviors of MNPs should be well established.

Furthermore, significant progress has been made in utilizing MH to enhance tumor immunogenicity and initiate anti-tumor immune responses. MH facilitates the release of tumor antigens and associated DAMPs, which are essential for immune activation. However, understanding the involvement of various immune cells in this process is crucial if we want to develop a more rational and comprehensive synergistic tumor treatment model that integrates MH and immunotherapy. Investigating the specific accumulation and effects of MNPs within distinct immune cell populations in the TME is imperative for us to achieving precise control over various types of immune cells. Gaining a comprehensive understanding of the intricate interplay between MNPs, resulting MH, and their interaction with the immune system is what is now needed to develop clinical strategies in this domain.

Moreover, research on MH has progressively shifted from macroscopic thermal ablation toward precise regulation of cellular functions. Through the rational integration of physical heat stimuli with specific biological signaling pathways, this approach has enabled substantial advances, particularly in the context of tumor therapy. At the tumor cell level, MH alters the “eat-me/don’t-eat-me” signal balance by bidirectionally regulating immune checkpoint molecules. It blocks the immune escape signal mediated by the CD47-SIRPα axis (the “don’t-eat-me” signal) and promotes CRT exposure on the cell surface (the “eat-me” signal), facilitating macrophage-mediated tumor cell phagocytosis. At the cell death pathway level, the synergistic effect of MH and ferroptosis overcomes tumor cell drug resistance and expands the scope of regulated cell death interventions. MH activates a comprehensive anti-tumor immune response, including macrophage polarization toward the M1 phenotype, DCs maturation and antigen cross-presentation, and restoration of T cell activity, enhancing their cytotoxic function. These studies confirm that MH is both an effective physical treatment for tumors and a powerful tool for regulating cell physiological functions. Its core value lies in the precise conversion of physical stimuli into biological effects, offering a novel approach to tumor therapy.

Magnetic hyperthermia is more appropriately defined as a regulatory rather than purely ablative technology because of a fundamental shift in both its mode of action and its biological consequences. Conventional hyperthermia primarily operates at the macroscopic or tissue level, relying on bulk temperature elevation to induce irreversible thermal damage and nonspecific cell ablation. In contrast, when magnetic nanoparticles are internalized by cells, alternating magnetic field-induced heating occurs at the intracellular or even subcellular scale.

Accumulating evidence indicates that such confined heating does not necessarily cause overt thermal necrosis. Instead, it selectively perturbs the function of key organelles-most notably lysosomes-thereby activating stress-responsive signaling pathways, metabolic reprogramming, and immune-related molecular cascades. Consequently, the dominant biological outcome shifts from indiscriminate cell killing toward precise modulation of cellular states and signaling networks, supporting the classification of magnetic hyperthermia as a regulatory technology.

Based on our understanding of MH’s core value and the systematic analysis of *in vivo* thermal effects and immune regulation mechanisms, we anticipate that the next-generation intelligent MH will achieve significant advancements in the next decade. This system will leverage the multifunctional properties of magnetic nanostructures to optimize the potential of various treatment methods through the integration of multiple technical strategies. Future research must establish a comprehensive framework for translating basic mechanisms into applications, focusing on the following three key directions:

First, material and equipment optimization must focus on addressing the challenges of uniformity and long-term biocompatibility in the mass production of MNPs. Research should focus on developing new functional materials, such as biodegradable magnesium alloys and acid-responsive core-shell structures, to enhance clinical translation safety from the outset. Additionally, artificial intelligence models should be used to construct a correlation between material components, AMF parameters, and therapeutic effects, enabling precise regulation of the SAR within the therapeutic window, and ensuring equipment safety and treatment efficacy.

Second, to improve delivery efficiency, a synergistic system should be developed, integrating MNPs, targeting molecules, and external magnetic fields (both static and alternating). Imaging technologies, such as magnetic resonance, should be utilized for real-time guidance and dynamic monitoring of the treatment process, overcoming technical challenges in deep tissue targeting and on-demand drug release. Additionally, the magnetic field-imaging combined delivery mode should be innovated to enhance treatment accuracy and controllability through multi-technology integration, advancing MH from basic research to clinical application.

Third, a deeper understanding of the TME's complexity is needed. Research should focus on analyzing the differential responses of cell populations, such as hepatoma cells and alveolar macrophages, to intracellular stimulation, and elucidate cell type-specific signaling activation mechanisms. Additionally, the release mechanisms of DAMPs, such as CRT exposure and ATP release, during MH-induced ICD should be systematically clarified. Research should also explore the molecular correlation between DAMPs and CD8^+^ T cell activation, as well as long-term adaptive immune memory formation, ultimately advancing the cross-scale theoretical framework of “MH stimulation-signal transduction-cell fate regulation”, providing core theoretical support for technology translation.

## Figures and Tables

**Table 1:** Application of magnetic nanomaterials in cancer therapy

Magnetic nanoparticles	Frequency (kHz)	Magnetic field (kA/m)	H x* f* (GA/m s)	SAR (W/g)	ILP (nHm^2^/kg)	Biological effect	Reference
Fe_3_O_4_/AIPH/DDP@PLGA	626	5.72	3.58	/	/	Magnetic controlled release	28
Zn_0.47_Mn_0.53_Fe_2_O_4_	389	19.5	7.59	/	/	Image-guided magnetic hyperthermia	33
Zn_0.4_Fe_2.6_O_4_ nanocubes	320	58.4	18.69	/	4.23	*In vivo* tumor magnetic hyperthermia	34
Iridium(III) modified MnFe_2_O_4_	274.5-598.0	23.9	6.56-14.29	47.6-145.4	/	*In vivo* tumor magnetic hyperthermia	35
Ferrimagnetic vortex-domain iron oxide nanorings	365	30-46	10.95-16.79	3337	4.4	*In vivo* tumor magnetic hyperthermia	36
Core-shell CoFe_2_O_4_@MnFe_2_O_4_	577	1.35	0.78	/	/	*In vivo* tumor magnetic hyperthermia	37
Fe@Fe_3_O_4_ core-shell nanoparticle	150	11.1-14.3	1.67-2.15	/	/	*In vivo* tumor magnetic hyperthermia	38
Core-shell Zn-CoFe_2_O_4_@Zn-MnFe_2_O_4_	-	1.7	-	321.44	-	*In vivo* tumor magnetic hyperthermia	39
Fe_3_O_4_@Au nanostars	470	14.3	6.72	-	-	Synergistic magnetic hyperthermia-photothermal therapy	40
CuFe_2_O_4_/SrTiO_3_	480	1.35	0.65	-	-	Magnetic hyperthermia-electric cascade for synergistic therapy	41
Fe_3_O_4_@Au@Cu_2-x_S trimers	110-300	24	2.6-7.2	250	-	Tri-modal therapy combining magnetic hyperthermia, photothermal therapy	42
Iron oxide nanoflowers @ CuS hybrids (IONF@CuS)	471	14.3	6.72	350	-	Tri-therapy combining magnetic hyperthermia, photothermal therapy	43

**Table 2:** Summary of several parameters, including SAR and ILP values of the Fe_3_O_4_ nanomaterials

	Core size (nm)	Hydrodynamic size (nm)	H (kA/m)	*f* (kHz)	SAR (W/g)	ILP (nHm^2^/kg)	References
Fe_3_O_4_	10	-	15	300	168	2.49	55
Fe_3_O_4_	15	-	37.3	500	450	0.65	55
Fe_3_O_4_	5	-	24.5	400	180	0.75	56
Fe_3_O_4_	10	-	-	-	130	0.54	56
Fe_3_O_4_	14	-	24.5	400	447	1.86	56
Fe_3_O_4_	12.5	-	-	-	200	0.83	56
Fe_3_O_4_	9	18	27	400	367	1.26	57
Fe_3_O_4_	9	21	27	400	332	1.14	57
Fe_3_O_4_	9	27	27	400	267	0.92	57
Fe_3_O_4_	19	25	27	400	930	3.19	57
Fe_3_O_4_	19	29	27	400	686	2.35	57
Fe_3_O_4_	19	35	27	400	535	1.83	57

**Table 3:** Comparative analysis of conductive nanostructures for eddy-current hyperthermia

Material system	Conductivity/geometry	Field parameters (H, *f*)	SAR	Proposed mechanism	Reference
Fe_3_O_4_@Au core-shell	spherical	H = 8 kA/m, *f* = 20 kHz	30.9 W/g	Hybrid: hysteresis (core) + eddy currents (shell)	58
rGO- Fe_3_O_4_ composites	2d sheets with NPs (µm lateral)	H = 10 kA/m, *f* = 200 kHz	400 W/g	Eddy currents in rgo sheets + minor hysteresis from Fe_3_O_4_	59
Gain liquid metal nps	spherical (100–500 nm)	H = 30 kA/m, *f* = 100 kHz	1500 W/g	Dominant eddy currents; deformable interface enhances cellular uptake	60, 61
Conductive polymer (PEDOT:PSS)-Fe_3_O_4_	nanocomposite films/particles	H = 9.9 kA/m, *f* = 322 kHz	100 W/g	Ohmic losses in polymer matrix; interfacial polarization	62

**Table 4:** Ten-year timeline of eddy-current magnetic hyperthermia development

Time	Core Materials	Key Breakthroughs	Representative Significance
2014-2016	Gold nanoshells, gold nanocages	Demonstrated that non-magnetic noble metal nanostructures can generate significant heating under AMF *via* eddy currents; heating efficiency scales with the square of the diameter	Only magnetic materials are suitable for magnetic hyperthermia; established conductivity and geometry as key design parameters
2017-2018	Fe_3_O_4_@Au core–shell, magnetoconductive hybrids	Developed synergistic systems combining a magnetic core with a conductive shell, enabling magnetic targeting, hysteresis loss, and eddy-current loss simultaneously	Overcame the weak magnetic responsiveness of pure metallic structures; opened the direction of multi-mechanism cooperative heating platforms
2019-2020	Carbon nanotubes, reduced graphene oxide composites	Exploited high electrical conductivity and anisotropic geometries; achieved ohmic losses in conductive networks	Introduced 2D materials and carbon-based conductive networks into magnetic hyperthermia, significantly enhancing mass-normalized heating efficiency
2021-2022	Gallium-indium liquid metal nanoparticles	Combined ultra-high conductivity with room-temperature fluidity; deformable interfaces improved cellular uptake	Pioneered liquid metals for magnetic hyperthermia, offering fluidic interfaces and record-high heating performance
2023--2026	Biodegradable magnetoconductive composites	Focused on biodegradability, clinical translation, and theranostic integration; combined MRI/CT contrast with controlled degradation profiles	Shifted from material-performance-driven to clinical-needs-driven design, emphasizing safety, imaging compatibility, and multifunctionality

## References

[1] Gilchrist RK, Medal R, Shorey WD, Hanselman RC, Parrott JC, Taylor CB (1957). Selective inductive heating of lymph nodes. Ann Surg.

[2] Liu X, Zhang Y, Wang Y, Zhu W, Li G, Ma X (2020). Comprehensive understanding of magnetic hyperthermia for improving antitumor therapeutic efficacy. Theranostics.

[3] Hatakeyama H, Wu SY, Lyons YA, Pradeep S, Wang W, Huang Q (2016). Role of CTGF in sensitivity to hyperthermia in ovarian and uterine cancers. Cell Rep.

[4] Jordan A, Wust P, Scholz R, Tesche B, Fähling H, Mitrovics T (1996). Cellular uptake of magnetic fluid particles and their effects on human adenocarcinoma cells exposed to AC magnetic fields *in vitro*. Int J Hyperthermia.

[5] Xiong L, Liang B, Yu K (2025). Magnetic hyperthermia in oncology: Nanomaterials-driven combinatorial strategies for synergistic therapeutic gains. Materials Today Bio.

[6] Ceccarelli MC, Paravizzini G, Marino A, Gigante G, Carmignani A, Catalano F (2025). Thermo-magnetic induction of pro-inflammatory microglia: A lipid-based nanovector strategy for glioblastoma immunotherapy. ACS Appl Mater Inter.

[7] Sadhukha T, Wiedmann TS, Panyam J (2013). Inhalable magnetic nanoparticles for targeted hyperthermia in lung cancer therapy. Biomaterials.

[8] Sharma SK, Shrivastava N, Rossi F, Tung LD, Thanh NTK (2019). Nanoparticles-based magnetic and photo induced hyperthermia for cancer treatment. Nano Today.

[9] Du Y, Liu XL, Liang Q, Liang XJ, Tian J (2019). Optimization and design of magnetic ferrite nanoparticles with uniform tumor distribution for highly sensitive MRI/MPI performance and improved magnetic hyperthermia therapy. Nano Lett.

[10] Laurent S, Dutz S, Häfeli UO, Mahmoudi M (2011). Magnetic fluid hyperthermia: Focus on superparamagnetic iron oxide nanoparticles. Adv Colloid Interface Sci.

[11] Maier-Hauff K, Ulrich F, Nestler D, Niehoff H, Wust P, Thiesen B (2011). Efficacy and safety of intratumoral thermotherapy using magnetic iron-oxide nanoparticles combined with external beam radiotherapy on patients with recurrent glioblastoma multiforme. J Neuro-Oncol.

[12] Jose J, Kumar R, Harilal S, Mathew GE, Parambi DGT, Prabhu A (2020). Magnetic nanoparticles for hyperthermia in cancer treatment: an emerging tool. Environ Sci Pollut Res Int.

[13] Fortin JP, Gazeau F, Wilhelm C (2008). Intracellular heating of living cells through Néel relaxation of magnetic nanoparticles. Eur Biophys J.

[14] Jang JT, Lee J, Seon J, Ju E, Kim M, Kim YI (2018). Giant magnetic heat induction of magnesium-doped γ-Fe_2_O_3_ superparamagnetic nanoparticles for completely killing tumors. Adv Mater.

[15] Das P, Salvioni L, Malatesta M, Vurro F, Mannucci S, Gerosa M (2020). Colloidal polymer-coated Zn-doped iron oxide nanoparticles with high relaxivity and specific absorption rate for efficient magnetic resonance imaging and magnetic hyperthermia. J Colloid Interface Sci.

[16] Ding Q, Liu DF, Guo DW, Yang F, Pang XY, Che BRE (2017). Shape-controlled fabrication of magnetite silver hybrid nanoparticles with high performance magnetic hyperthermia. Biomaterials.

[17] Das R, Rinaldi-Montes N, Alonso J, Amghouz Z, Garaio E, García JA (2016). Boosted hyperthermia therapy by combined AC magnetic and photothermal exposures in Ag/Fe_3_O_4_ nanoflowers. Acs Appl Mater Interfaces.

[18] de la Encarnación C, Jungwirth F, Vila-Liarte D, Renero-Lecuna C, Kavak S, Orue I (2023). Hybrid core-shell nanoparticles for cell-specific magnetic separation and photothermal heating. J Mater Chem B.

[19] Yue Q, Zhang Y, Wang C, Wang XQ, Sun ZK, Hou XF (2015). Magnetic yolk-shell mesoporous silica microspheres with supported Au nanoparticles as recyclable high-performance nanocatalysts. J Mater Chem A.

[20] Vijayan V, Sundaram A, Vasukutty A, Bardhan R, Uthaman S, Park IK (2023). Tumor-targeting cell membrane-coated nanorings for magnetic-hyperthermia-induced tumor ablation. Biomater Sci.

[21] Chen YH, Cheng CH, Chang WJ, Lin YC, Lin FH, Lin JC (2016). Studies of magnetic alginate-based electrospun matrices crosslinked with different methods for potential hyperthermia treatment. Mater Sci Eng C.

[22] Remya NS, Syama S, Sabareeswaran A, Mohanan PV (2016). Toxicity, toxicokinetics and biodistribution of dextran stabilized Iron oxide Nanoparticles for biomedical applications. Int J Pharm.

[23] Pham XN, Nguyen TP, Pham TN, Tran TTN, Tran TVT (2016). Synthesis and characterization of chitosan-coated magnetite nanoparticles and their application in curcumin drug delivery. Adv Nat Sci-Nanosci.

[24] Bilal M, Iqbal HMN, Adil SF, Shaik MR, Abdelgawad A, Hatshan MR (2022). Surface-coated magnetic nanostructured materials for robust bio-catalysis and biomedical applications-A review. J Adv Res.

[25] Sindhwani S, Syed AM, Ngai J, Kingston BR, Maiorino L, Rothschild J (2020). The entry of nanoparticles into solid tumours. Nat Mater.

[26] Yuan H, Li X, Yu M, Cao Y, Wu L, Shi S (2025). Injectable SF-platform orchestrates GPX4-targeted ferroptosis-autophagy-immunogenic circuit for overcoming oxidative resistance in triple-negative breast cancer. Theranostics.

[27] Dai P, Zhu W, Yan B, Miao Y, Hu S, Gao X (2021). Regulation of ID4 *in vivo* for efficient magnetothermal therapy of breast cancer. Adv Ther.

[28] Wu L, Su J, Cao Y, Yuan H, Li Z, Ren J (2026). Dual-targeting nuclear and mitochondrial DNA damage drives immunogenic activation via PANoptosis for synergistic magneto-thermodynamic-chemotherapy. Biomaterials.

[29] Yan B, Wang S, Liu C, Wen N, Li H, Zhang Y (2022). Engineering magnetic nano-manipulators for boosting cancer immunotherapy. J Nanobiotechnol.

[30] Liu C, Yan B, Wen N, Li W, Wang J, Li H (2023). Engineering energy-responsive magnetic nanomaterials to improve the efficacy of dendritic cell-based immunotherapy. Adv Ther.

[31] Hu M, Zhang J, Kong L, Yu Y, Hu Q, Yang T (2021). Immunogenic hybrid nanovesicles of liposomes and tumor-derived nanovesicles for cancer immunochemotherapy. ACS Nano.

[32] Shaterabadi Z, Nabiyouni G, Soleymani M (2018). Physics responsible for heating efficiency and self-controlled temperature rise of magnetic nanoparticles in magnetic hyperthermia therapy. Prog Biophys Mol Bio.

[33] Zhang Z-Q, Song S-C (2016). Thermosensitive/superparamagnetic iron oxide nanoparticle-loaded nanocapsule hydrogels for multiple cancer hyperthermia. Biomaterials.

[34] Chen X, Wang H, Shi J, Chen Z, Wang Y, Gu S (2023). An injectable and active hydrogel induces mutually enhanced mild magnetic hyperthermia and ferroptosis. Biomaterials.

[35] Shen J, Rees TW, Zhou Z, Yang S, Ji L, Chao H (2020). A mitochondria-targeting magnetothermogenic nanozyme for magnet-induced synergistic cancer therapy. Biomaterials.

[36] Liu X, Zheng J, Sun W, Zhao X, Li Y, Gong N (2019). Ferrimagnetic vortex nanoring-mediated mild magnetic hyperthermia imparts potent immunological effect for treating cancer metastasis. ACS Nano.

[37] Pan J, Hu P, Guo Y, Hao J, Ni D, Xu Y (2020). Combined magnetic hyperthermia and immune therapy for primary and metastatic tumor treatments. ACS Nano.

[38] Koo C, Hong H, Im PW, Kim H, Lee C, Jin X (2020). Magnetic and near-infrared derived heating characteristics of dimercaptosuccinic acid coated uniform Fe@Fe_3_O_4_ core-shell nanoparticles. Nano Convergence.

[39] Pan J, Xu Y, Wu Q, Hu P, Shi J (2021). Mild magnetic hyperthermia-activated innate immunity for liver cancer therapy. J Am Chem Soc.

[40] Espinosa A, Reguera J, Curcio A, Muñoz-Noval Á, Kuttner C, Van de Walle A (2020). Janus magnetic-plasmonic nanoparticles for magnetically guided and thermally activated cancer therapy. Small.

[41] Yuan X, Kang Y, Li R, Niu G, Shi J, Yang Y (2025). Magnetically triggered thermoelectric heterojunctions with an efficient magnetic-thermo-electric energy cascade conversion for synergistic cancer therapy. Nat Commun.

[42] Fiorito S, Soni N, Silvestri N, Brescia R, Gavilán H, Conteh JS (2022). Fe_3_O_4_@Au@Cu_2-x_S heterostructures designed for trimodal therapy: Photomagnetic hyperthermia and ^64^cu radioinsertion. Small.

[43] Curcio A, Silva AKA, Cabana S, Espinosa A, Baptiste B, Menguy N (2019). Iron oxide nanoflowers@CuS hybrids for cancer tri-therapy: interplay of photothermal therapy, magnetic hyperthermia and photodynamic therapy. Theranostics.

[44] Gavilán H, Avugadda SK, Fernández-Cabada T, Soni N, Cassani M, Mai BT (2021). Magnetic nanoparticles and clusters for magnetic hyperthermia: optimizing their heat performance and developing combinatorial therapies to tackle cancer. Chem Soc Rev.

[45] Rosensweig RE (2002). Heating magnetic fluid with alternating magnetic field. J Magn Magn Mater.

[46] Blanco-Andujar C, Ortega D, Southern P, Pankhurst QA, Thanh NT (2015). High performance multi-core iron oxide nanoparticles for magnetic hyperthermia: microwave synthesis, and the role of core-to-core interactions. Nanoscale.

[47] Meredith RF, Brezovich IA, Weppelmann B, Henderson RA, Brawner WR, Kwapien RP (1989). Ferromagnetic thermoseeds: Suitable for an afterloading interstitial implant. Int J Radiat Oncol.

[48] Thiesen B, Jordan A (2008). Clinical applications of magnetic nanoparticles for hyperthermia. Int J Hyperthermia.

[49] Wust P, Gneveckow U, Johannsen M, Böhmer D, Henkel T, Kahmann F (2006). Magnetic nanoparticles for interstitial thermotherapy-feasibility, tolerance and achieved temperatures. Int J Hyperthermia.

[50] Di Corato R, Béalle G, Kolosnjaj-Tabi J, Espinosa A, Clément O, Silva AK (2015). Combining magnetic hyperthermia and photodynamic therapy for tumor ablation with photoresponsive magnetic liposomes. ACS Nano.

[51] Shen Y, Li X, Huang H, Lan Y, Gan L, Huang J Embedding Mn2+ in polymer coating on rod-like cellulose nanocrystal to integrate MRI and photothermal function. Carbohydrate Polymers. 297: 120061.

[52] Li X, Li W, Wang M, Liao Z (2021). Magnetic nanoparticles for cancer theranostics: Advances and prospects. J Control Release.

[53] Chen Z, Wu C, Zhang Z, Wu W, Wang X, Yu Z (2018). Synthesis, functionalization, and nanomedical applications of functional magnetic nanoparticles. Chin Chem Lett.

[54] Lim J, Lanni C, Evarts ER, Lanni F, Tilton RD, Majetich SA (2011). Magnetophoresis of nanoparticles. ACS Nano.

[55] Pradhan P, Giri J, Samanta G, Sarma HD, Mishra KP, Bellare J (2007). Comparative evaluation of heating ability and biocompatibility of different ferrite-based magnetic fluids for hyperthermia application. J Biomed Mater Res B Appl Biomater.

[56] Gonzales-Weimuller M, Zeisberger M, Krishnan KM (2009). Size-dependant heating rates of iron oxide nanoparticles for magnetic fluid hyperthermia. J Magn Magn Mater.

[57] Liu XL, Fan HM, Yi JB, Yang Y, Choo ESG, Xue JM (2012). Optimization of surface coating on Fe3O_4_ nanoparticles for high performance magnetic hyperthermia agents. J Mater Chem.

[58] Brennan G, Bergamino S, Pescio M, Tofail SAM, Silien C (2020). The effects of a varied gold shell thickness on iron oxide nanoparticle cores in magnetic manipulation, T_1_ and T_2_ MRI contrasting, and magnetic hyperthermia. Nanomaterials.

[59] Zhang Y, Lv X, Zhang Y, Jiang Z, Gong C (2021). Tunable microwave absorbing property of RGO/Fe_3_O_4_/SiO_2_ nanocomposites by effective regulation of eddy current effect. J Appl Phys.

[60] Yang N, Li W, Gong F, Cheng L, Dong Z, Bai S (2020). Injectable nonmagnetic liquid metal for eddy-thermal ablation of tumors under alternating magnetic field. Small Methods.

[61] Wang D, Xie W, Gao Q, Yan H, Zhang J, Lu J (2019). Non-magnetic injectable implant for magnetic field-driven thermochemotherapy and dual stimuli-responsive drug delivery: transformable liquid metal hybrid platform for cancer theranostics. Small.

[62] Stefan N, Visan AI, Grumezescu V, Kuncser V, Kuncser A, Iacob N (2024). MAPLE deposition of hybrid PLGA-Fe_3_O_4_- Cypress-PEDOT: PSS coatings. Giant.

[63] Shaterabadi Z, Nabiyouni G, Soleymani M (2018). Physics responsible for heating efficiency and self-controlled temperature rise of magnetic nanoparticles in magnetic hyperthermia therapy. Prog Biophys Mol Biol.

[64] Kim K, Park Y-G, Hyun BG, Choi M, Park J-U (2019). Recent advances in transparent electronics with stretchable forms. Adv Mater.

[65] Mehdaoui B, Meffre A, Lacroix LM, Carrey J, Lachaize S, Gougeon M (2010). Large specific absorption rates in the magnetic hyperthermia properties of metallic iron nanocubes. J Magn Magn Mater.

[66] Dennis CL, Ivkov R (2013). Physics of heat generation using magnetic nanoparticles for hyperthermia. Int J Hyperthermia.

[67] Kolhatkar AG, Jamison AC, Litvinov D, Willson RC, Lee TR (2013). Tuning the magnetic properties of nanoparticles. Int J Mol Sci.

[68] Deatsch AE, Evans BA (2014). Heating efficiency in magnetic nanoparticle hyperthermia. J Magn Magn Mater.

[69] Guardia P, Di Corato R, Lartigue L, Wilhelm C, Espinosa A, Garcia-Hernandez M (2012). Water-soluble iron oxide nanocubes with high values of dpecific sbsorption rate for cancer cell hyperthermia treatment. ACS Nano.

[70] Hergt R, Dutz S, Müller R, Zeisberger M (2006). Magnetic particle hyperthermia: nanoparticle magnetism and materials development for cancer therapy. J Phys-Condens Mat.

[71] Dutz S, Hergt R (2014). Magnetic particle hyperthermia-a promising tumour therapy?. Nanotechnology.

[72] Rezaei B, Yari P, Sanders SM, Wang H, Chugh VK, Liang S (2024). Magnetic nanoparticles: A review on synthesis, characterization, functionalization, and biomedical applications. Small.

[73] Noh S-h, Na W, Jang J-t, Lee J-H, Lee EJ, Moon SH (2012). Nanoscale magnetism control via surface and exchange anisotropy for optimized ferrimagnetic hysteresis. Nano Lett.

[74] Liu XL, Yang Y, Ng CT, Zhao LY, Zhang Y, Bay BH (2015). Magnetic vortex nanorings: A new class of hyperthermia agent for highly efficient *in vivo* regression of tumors. Adv Mater.

[75] Zhao C-X, Liu J-N, Li B-Q, Ren D, Chen X, Yu J (2020). Multiscale construction of bifunctional electrocatalysts for long-lifespan rechargeable zinc-air batteries. Adv Funct Mater.

[76] Lee J-H, Huh Y-M, Jun Y-w, Seo J-w, Jang J-t, Song H-T (2007). Artificially engineered magnetic nanoparticles for ultra-sensitive molecular imaging. Nat Med.

[77] Jang J-t, Nah H, Lee J-H, Moon SH, Kim MG, Cheon J (2009). Critical enhancements of MRI contrast and hyperthermic effects by dopant-controlled magnetic nanoparticles. Angew Chem Int Ed.

[78] Lee J-H, Jang J-t, Choi J-s, Moon SH, Noh S-h, Kim J-w (2011). Exchange-coupled magnetic nanoparticles for efficient heat induction. Nat Nanotechnol.

[79] Zhang Y, Ding H, Liu Y, Pan S, Luo Y, Li G (2012). Facile one-step synthesis of plasmonic/magnetic core/shell nanostructures and their multifunctionality. Journal of Materials Chemistry.

[80] Curcio A, Silva AKA, Cabana S, Espinosa A, Baptiste B, Menguy N (2019). Iron Oxide Nanoflowers @ CuS Hybrids for Cancer Tri-Therapy: Interplay of Photothermal Therapy, Magnetic Hyperthermia and Photodynamic Therapy. Theranostics.

[81] Kakwere H, Materia ME, Curcio A, Prato M, Sathya A, Nitti S (2018). Dually responsive gold-iron oxide heterodimers: merging stimuli-responsive surface properties with intrinsic inorganic material features. Nanoscale.

[82] Liu X, Yan B, Li Y, Ma X, Jiao W, Shi K (2020). Graphene oxide-grafted magnetic nanorings mediated magnetothermodynamic therapy favoring reactive oxygen species-related immune response for enhanced antitumor efficacy. ACS Nano.

[83] Gu Y, Piñol R, Moreno-Loshuertos R, Brites CDS, Zeler J, Martínez A (2023). Local temperature increments and induced cell death in intracellular magnetic hyperthermia. ACS Nano.

[84] Qiu Y, Tong S, Zhang L, Sakurai Y, Myers DR, Hong L (2017). Magnetic forces enable controlled drug delivery by disrupting endothelial cell-cell junctions. Nat Commun.

[85] Liu Y-L, Chen D, Shang P, Yin D-C (2019). A review of magnet systems for targeted drug delivery. J Control Release.

[86] Harris M, Ahmed H, Barr B, LeVine D, Pace L, Mohapatra A (2017). Magnetic stimuli-responsive chitosan-based drug delivery biocomposite for multiple triggered release. Int J Biol Macromol.

[87] Liu J, Cabral H, Mi P (2024). Nanocarriers address intracellular barriers for efficient drug delivery, overcoming drug resistance, subcellular targeting and controlled release. Adv Drug Deliv Rev.

[88] Mohammad F, Bwatanglang IB, Al-Lohedan HA, Shaik JP, Moosavi M, Dahan WM (2023). Magnetically controlled drug delivery and hyperthermia effects of core-shell Cu@Mn_3_O_4_ nanoparticles towards cancer cells *in vitro*. Int J Biol Macromol.

[89] Chen W, Cheng C-A, Zink JI (2019). Spatial, temporal, and dose control of drug delivery using noninvasive magnetic stimulation. ACS Nano.

[90] Liu X, Zhang Y, Guo Y, Jiao W, Gao X, Lee WSV (2021). Electromagnetic field-programmed magnetic vortex nanodelivery system for efficacious cancer therapy. Adv Sci.

[91] Dai L, Jiao W, Li J, He Z, Lu Q, Zheng C (2025). Intracellular magnetic nanodelivery of H_2_S and heat synergize to reshape the tumor immune microenvironment. Adv Healthc Mater.

[92] Ye Z, Yan B, Li H, Tang Q, Yuan K, Hou J (2025). Dual-responsive magnetic vortex nanorings co-deliver lenvatinib and localized heat for synergistic activation of antitumor immunity. Acta Biomater.

[93] Maier-Hauff K, Rothe R, Scholz R, Gneveckow U, Wust P, Thiesen B (2007). Intracranial thermotherapy using magnetic nanoparticles combined with external beam radiotherapy: results of a feasibility study on patients with glioblastoma multiforme. J Neurooncol.

[94] Johannsen M, Gneveckow U, Taymoorian K, Thiesen B, Waldöfner N, Scholz R (2007). Morbidity and quality of life during thermotherapy using magnetic nanoparticles in locally recurrent prostate cancer: results of a prospective phase I trial. Int J Hyperthermia.

[95] Yu K, Zhou H, Xu Y, Cao Y, Zheng Y, Liang B (2023). Engineering a triple-functional magnetic gel driving mutually-synergistic mild hyperthermia-starvation therapy for osteosarcoma treatment and augmented bone regeneration. J Nanobiotechnol.

[96] Bai S, Hou S, Chen T, Ma X, Gao C, Wu A (2024). Magnetic nanoparticle-mediated hyperthermia: From heating mechanisms to cancer theranostics. Innov Mater.

[97] Yanase M, Shinkai M, Honda H, Wakabayashi T, Yoshida J, Kobayashi T (1998). Antitumor immunity induction by intracellular hyperthermia using magnetite cationic liposomes. Jpn J Cancer Res.

[98] Beola L, Asín L, Fratila RM, Herrero V, de la Fuente JM, Grazú V (2018). Dual role of magnetic nanoparticles as intracellular hotspots and extracellular matrix disruptors triggered by magnetic hyperthermia in 3D cell culture models. ACS Appl Mater Interfaces.

[99] Kroemer G, Galassi C, Zitvogel L, Galluzzi L (2022). Immunogenic cell stress and death. Nat Immunol.

[100] Li Z, Lai X, Fu S, Ren L, Cai H, Zhang H (2022). Immunogenic cell death activates the tumor immune microenvironment to boost the immunotherapy efficiency. Adv Sci.

[101] Galluzzi L, Vitale I, Warren S, Adjemian S, Agostinis P, Martinez AB (2020). Consensus guidelines for the definition, detection and interpretation of immunogenic cell death. J Immunother Cancer.

[102] Yan B, Liu C, Wang S, Li H, Jiao J, Lee WSV (2022). Magnetic hyperthermia induces effective and genuine immunogenic tumor cell death with respect to exogenous heating. J Mater Chem B.

[103] Zhang Y, Zhang Y, Li J, Liang C, Shi K, Wang S (2024). Impact of nanoheater subcellular localization on the antitumor immune efficacy of magnetic hyperthermia. Nano Today.

[104] Cheng X, Xu J, Cui Y, Liu J, Chen Y, He C (2025). Nanovesicles for lipid metabolism reprogram-enhanced ferroptosis and magnetotherapy of refractory tumors and inhibiting metastasis with activated innate immunity. ACS Nano.

[105] Yan B, Liu C, Li H, Wen N, Jiao W, Wang S (2023). Reversal of HMGA1-mediated immunosuppression synergizes with immunogenic magnetothermodynamic for improved hepatocellular carcinoma therapy. ACS Nano.

[106] Zhang Y, Zhang Z (2020). The history and advances in cancer immunotherapy: understanding the characteristics of tumor-infiltrating immune cells and their therapeutic implications. Cell Mol Immunol.

[107] Ge J, Yang N, Yang Y, Yu H, Yang X, Wang Y (2023). The combination of eddy thermal effect of biodegradable magnesium with immune checkpoint blockade shows enhanced efficacy against osteosarcoma. Bioact Mater.

[108] Wang Y, Xiang Y, Xin VW, Wang X-W, Peng X-C, Liu X-Q (2020). Dendritic cell biology and its role in tumor immunotherapy. J Hematol.

[109] Del Prete A, Salvi V, Soriani A, Laffranchi M, Sozio F, Bosisio D (2023). Dendritic cell subsets in cancer immunity and tumor antigen sensing. Cell Mol Immunol.

[110] Wculek SK, Cueto FJ, Mujal AM, Melero I, Krummel MF, Sancho D (2020). Dendritic cells in cancer immunology and immunotherapy. Nat Rev Immunol.

[111] Liu J, Zhang X, Cheng Y, Cao X (2021). Dendritic cell migration in inflammation and immunity. Cell Mol Immunol.

[112] Chang Z-g, Yang L-y, Wang W, Peng J-x, Huang G-w, Tao Y-m (2005). Determination of high mobility group A1 (HMGA1) expression in hepatocellular carcinoma: A potential prognostic marker. Digest Dis Sci.

[113] Bied M, Ho WW, Ginhoux F, Blériot C (2023). Roles of macrophages in tumor development: a spatiotemporal perspective. Cell Mol Immunol.

[114] Noy R, Pollard Jeffrey W (2014). Tumor-associated macrophages: from mechanisms to therapy. Immunity.

[115] Jiang H, Fu H, Guo Y, Hu P, Shi J (2022). Evoking tumor associated macrophages by mitochondria-targeted magnetothermal immunogenic cell death for cancer immunotherapy. Biomaterials.

[116] Wang S, Jiao W, Yan B, Liu X, Tang Q, Zhang Y (2024). Intracellular magnetic hyperthermia enables concurrent down-regulation of CD47 and SIRPα to potentiate antitumor immunity. Nano Lett.

[117] Oliveira G, Wu CJ (2023). Dynamics and specificities of T cells in cancer immunotherapy. Nat Rev Cancer.

[118] de Visser KE, Joyce JA (2023). The evolving tumor microenvironment: From cancer initiation to metastatic outgrowth. Cancer Cell.

[119] Carter TJ, Agliardi G, Lin F-Y, Ellis M, Jones C, Robson M (2021). Potential of magnetic hyperthermia to stimulate localized immune activation. Small.

[120] Toraya-Brown S, Sheen MR, Zhang P, Chen L, Baird JR, Demidenko E (2014). Local hyperthermia treatment of tumors induces CD8^+^ T cell-mediated resistance against distal and secondary tumors. Nanomedicine: NBM.

[121] Darvin P, Toor SM, Nair VS, Elkord E (2018). Immune checkpoint inhibitors: recent progress and potential biomarkers. Exp Mol Med.

[122] Marin-Acevedo JA, Dholaria B, Soyano AE, Knutson KL, Chumsri S, Lou YY (2018). Next generation of immune checkpoint therapy in cancer: new developments and challenges. J Hematol Oncol.

[123] Doroshow DB, Bhalla S, Beasley MB, Sholl LM, Kerr KM, Gnjatic S (2021). PD-L1 as a biomarker of response to immune-checkpoint inhibitors. Nat Rev Clin Oncol.

[124] Ahn E, Araki K, Hashimoto M, Li WY, Riley JL, Cheung J (2018). Role of PD-1 during effector CD8 T cell differentiation. Proc Natl Acad Sci USA.

[125] Pang K, Shi Z-D, Wei L-Y, Dong Y, Ma Y-Y, Wang W (2023). Research progress of therapeutic effects and drug resistance of immunotherapy based on PD-1/PD-L1 blockade. Drug Resist.

[126] Gao M, Feng K, Zhang X, Ruan Y, Zhao G, Liu H (2023). Nanoassembly with self-regulated magnetic thermal therapy and controlled immuno-modulating agent release for improved immune response. J Control Release.

[127] Waldman AD, Fritz JM, Lenardo MJ (2020). A guide to cancer immunotherapy: from T cell basic science to clinical practice. Nat Rev Immunol.

[128] Mullard A (2016). The cancer vaccine resurgence. Nat Rev Drug Discov.

[129] Sheng J, Liu Y, Ding H, Wu L, Liu L, Si G (2023). Magnetic delivery of antigen-loaded magnetic liposomes for active lymph node targeting and enhanced anti-tumor immunity. Adv Healthc Mater.

[130] Sun Z, Chu Y, Xiao J, Yang Y, Meng F, Wang X (2023). Enhanced systemic tumor suppression by *in situ* vaccine combining radiation and OX40 agonist with CpG therapy. J Transl Med.

[131] Saxena M, van der Burg SH, Melief CJM, Bhardwaj N (2021). Therapeutic cancer vaccines. Nat Rev Cancer.

[132] Lin MJ, Svensson-Arvelund J, Lubitz GS, Marabelle A, Melero I, Brown BD (2022). Cancer vaccines: the next immunotherapy frontier. Nat Cancer.

[133] Lafuente-Gómez N, de Lázaro I, Dhanjani M, García-Soriano D, Sobral MC, Salas G (2023). Multifunctional magnetic nanoparticles elicit anti-tumor immunity in a mouse melanoma model. Mater Today Bio.

[134] Jin H, Qian Y, Dai Y, Qiao S, Huang C, Lu L (2016). Magnetic enrichment of dendritic cell vaccine in lymph node with fluorescent-magnetic nanoparticles enhanced cancer immunotherapy. Theranostics.

[135] Huang L, Liu Z, Wu C, Lin J, Liu N (2023). Magnetic nanoparticles enhance the cellular immune response of dendritic cell tumor vaccines by realizing the cytoplasmic delivery of tumor antigens. Bioeng Transl Med.

[136] Chen F, Li T, Zhang H, Saeed M, Liu X, Huang L (2023). Acid-ionizable iron nanoadjuvant augments sting activation for personalized vaccination immunotherapy of cancer. Adv Mater.

[137] Manescu V, Antoniac I, Paltanea G, Nemoianu IV, Mohan AG, Antoniac A (2024). Magnetic hyperthermia in glioblastoma multiforme treatment. International Journal of Molecular Sciences.

[138] Jiang Z, Li T, Cheng H, Zhang F, Yang X, Wang S (2021). Nanomedicine potentiates mild photothermal therapy for tumor ablation. Asian J Pharm Sci.

[139] Yi X, Duan QY, Wu FG (2021). Low-temperature photothermal therapy: Strategies and applications. Research.

[140] Kho ASK, Ooi EH, Foo JJ, Ooi ET (2024). Saline-infused radiofrequency ablation: A review on the key factors for a safe and reliable tumour treatment. IEEE Rev Biomed Eng.

[141] Cheng X, He C, Huang J, Li J, Hu Z, Wang L (2024). A tumor-homing nanoframework for synergistic microwave tumor ablation and provoking strong anticancer immunity against metastasis. ACS Nano.

[142] Shakeri-Zadeh A, Bulte JWM (2025). Imaging-guided precision hyperthermia with magnetic nanoparticles. Nat Rev Bioeng.

[143] Laha SS, Thorat ND, Singh G, Sathish CI, Yi J, Dixit A (2022). Rare-earth doped iron oxide nanostructures for cancer theranostics: Magnetic hyperthermia and magnetic resonance imaging. Small.

[144] Carlton H, Salimi M, Arepally N, Bentolila G, Sharma A, Bibic A (2025). Ranking magnetic colloid performance for magnetic particle imaging and magnetic particle hyperthermia. Adv Funct Mater.

[145] Buchholz O, Sajjamark K, Franke J, Wei H, Behrends A, Münkel C (2024). *In situ* theranostic platform combining highly localized magnetic fluid hyperthermia, magnetic particle imaging, and thermometry in 3D. Theranostics.

